# Hybridndiff-UQ: Uncertainty quantification for hybrid neural differentiable modeling

**DOI:** 10.1016/j.taml.2025.100609

**Published:** 2026-03-10

**Authors:** Deepak Akhare, Tengfei Luo, Jian-Xun Wang

**Affiliations:** a Department of Aerospace and Mechanical Engineering, University of Notre Dame, Notre Dame, IN, USA; b Department of Chemical and Biomolecular Engineering, University of Notre Dame, Notre Dame, IN, USA; c Sibley School of Mechanical and Aerospace Engineering, Cornell University, Ithaca, NY, USA

**Keywords:** Differentiable programming, Scientific machine learning, Grey box modeling, Uncertainty quantification, Scalable bayesian learning

## Abstract

The hybrid neural differentiable models mark a significant advancement in the field of scientific machine learning. These models, integrating numerical representations of known physics into deep neural networks, offer enhanced predictive capabilities and show great potential for data-driven modeling of complex physical systems. However, a critical and yet unaddressed challenge lies in the quantification of inherent uncertainties stemming from multiple sources. Addressing this gap, we introduce a novel method, uncertainty quantification for hybrid neural differentiable modeling, for effective and efficient uncertainty propagation and estimation in hybrid neural differentiable models, leveraging the strengths of deep ensemble Bayesian learning and nonlinear transformations. Specifically, our approach effectively discerns and quantifies both aleatoric uncertainties, arising from data noise, and epistemic uncertainties, resulting from model-form discrepancies and data sparsity. This is achieved within a Bayesian model averaging framework, where aleatoric uncertainties are modeled through hybrid neural models. The unscented transformation plays a pivotal role in enabling the flow of these uncertainties through the nonlinear functions within the hybrid model. In contrast, epistemic uncertainties are estimated using an ensemble of stochastic gradient descent trajectories. This approach offers a practical approximation to the posterior distribution of both the network parameters and the physical parameters. Notably, our framework is designed for simplicity in implementation and high scalability, making it suitable for parallel computing environments. The merits of the proposed method have been demonstrated through problems governed by both ordinary and partial differentiable equations.

## 1. Introduction

The evolving dynamics of computational science are driven by the advent of advanced numerical algorithms, enhanced computational infrastructures, and the proliferation of extensive datasets. This evolution has catalyzed the development of innovative methodologies to address multifaceted challenges associated with scientific modeling and predictive analytics. Within this transformative framework, scientific machine learning (SciML) emerges as a salient discipline, intricately blending the principles of traditional scientific modeling with the state-of-the-art machine learning techniques, especially in the context of handling voluminous data and advanced GPU computing. At its core, SciML represents a paradigm that seamlessly integrates the rigorous foundations of scientific principles with the adaptability and efficiency of machine learning methods. Such an integration not only amplifies the computational efficiency but often enhances the accuracy of predictions for complex scientific problems. Illustrative of the advancements within the SciML domain are methodologies like physics-informed neural networks (PINN) [1-3], neural operators [4-6], equation discovery techniques [7-9], and hybrid neural models [10-14].

In the expanding landscape of SciML, hybrid neural modeling emerges as a distinctive frontier that marries domain-specific knowledge with cutting-edge data-driven methodologies, striking a balance between deep-rooted scientific principles and the adaptability of modern machine learning (ML) techniques. While traditional scientific models, rooted in established physical laws and equations, are invaluable tools for understanding various physical phenomena, they often struggle when faced with complex systems whose underlying physics has not been fully understood or when managing extensive datasets. In contrast, deep neural networks (DNNs) are adept at deciphering intricate patterns from large-scale data, but their black-box nature can pose interpretability challenges and sometimes limits their generalization in out-of-sample scenarios. Combining the strengths of traditional scientific models with ML, hybrid techniques endeavor to address these challenges, leveraging the advantages of both worlds. Past effort in hybrid learning model tend to weakly integrate ML with physics-based numerical models [15,16]. Typically, an ML model is trained offline, and later incorporated into a standard numerical solver. Such strategies gained traction, especially in data-driven closure modeling for turbulence [17-19] or atmospheric flows [14,20]. A significant challenge, however, arises from the necessity for labeled data during intermediate phases—a requirement that is frequently unmet. Furthermore, when ML models and numerical solvers operate somewhat independently, the resulting hybrid system can sometimes exhibit instability, leading to unreliable and often inaccurate a posteriori predictions [21].

Recognizing these challenges, the prevailing consensus highlights the advantages of a more integrated approach – creating a unified hybrid model that promises not just to improve computational efficiency, but also yield more robust and accurate solutions. The paradigm of differentiable programming (DP) offers a pathway to this goal, enabling joint optimization of both ML and numerical components within a unified training environment. Recently, there has been a growing interest in developing differentiable solvers and hybrid neural models, which have demonstrated significant potential across various scientific fields [10-13,22-32]. For example, Kochkov et al. [10] integrated convolutional neural networks into a differentiable computational fluid dynamic (CFD) solver, facilitating more effective coarse graining. Similarly, Huang et al. [24] incorporated DNNs into a differentiable finite element solver, enabling the derivation of constitutive relations for nonlinear materials from indirect measurements. Wang and co-workers have pioneered differentiable hybrid neural models to efficiently emulate spatiotemporal physics of fluid [11], fluid-structure interaction [12], and composite materials manufacturing processes [13]. Their designs are deeply rooted in the profound relationship between neural network architectural components (e.g., convolution layers, residual connections) and the numerical representations of PDEs - a subject currently gaining traction in the ML community [33-37]. Building on these connections, they pointed out that traditional numerical solvers can be interpreted as unique neural network instances, where the convolutional kernels, recurrent structures, and residual connections are pre-determined entirely by the known physics and associated numerical schemes, rather than being learned from data [11]. Therefore, the differentiable hybrid neural models can be viewed as physics-integrated neural networks, where the known physics, represented by discretized PDE operators, are woven into the neural architecture, guided by the connection between numerical PDEs and neural architecture components [12].

Despite the promise of hybrid neural modeling in advancing computational science and engineering, addressing the intrinsic uncertainties in these models remains a significant concern. These models, while effectively integrating domain-specific knowledge with machine learning methodologies, are not immune to uncertainties from multiple sources. These uncertainties can be categorized into two types: aleatoric/data uncertainty, an irreducible variability that arises from inherent noise in data or measurements, and epistemic/model uncertainty, reducible uncertainty that stems from limited knowledge, insufficient data coverage, or model-form inadequacies. Aleatoric uncertainty is commonly encountered in noisy experimental observations, sensor errors, or stochastic initial conditions. In contrast, epistemic uncertainty often arises from missing or simplified physics (e.g., unmodeled phenomena, coarse discretization in PDEs) or the overparameterized nature of neural networks. For example, a PDE-based simulator using coarse-grained closure approximations in turbulence models introduces epistemic bias, while instrumentation noise in heat flux measurements results in aleatoric noise. In the context of hybrid neural models, aleatoric uncertainty can emerge from noisy training datasets, while the vast parameter landscape of neural networks and potential model-form discrepancy of physics-based models can induce epistemic uncertainty. In practice, these two uncertainty types interact and vary by application. In high-noise or high-variability data environments, aleatoric uncertainty tends to dominate, while in extrapolative or data-scarce scenarios, epistemic uncertainty becomes more significant—making it crucial to model both. The growing prominence of these issues has brought uncertainty quantification (UQ) in SciML into the spotlight [38-40]. As we know, UQ for DNNs is a long-standing challenge within the ML community, largely due to the intricate, nonlinear nature of DL models. The extremely high-dimensional parameter space of DL models make traditional Bayesian sampling techniques infeasible [41]. This limitation has directed researchers toward approximate Bayesian learning strategies such as variational inference [42,43], stochastic gradient MCMC [44,45], and Bayes by back-propagation [46]. In parallel, evidential deep learning [47,48], subspace inference [49-52] ensemble learning methods such as deep ensemble [53], snapshot ensemble [54], SWAG [55], and SeBayS [56] have emerged as effective tools for estimating uncertainty in DNNs. The posterior landscape, however, becomes even more intricate for hybrid neural models that blend physics-based numerical solvers with DNNs. The fusion of mathematical representations of physical phenomena with the inherent complexities of neural networks introduces multifaceted sources of uncertainty. How to quantify and propagate uncertainty for these differentiable hybrid neural models remains uncharted territory, indicating a significant gap in the current literature.

In this study, we present HybridNDiff-UQ, a novel probabilistic hybrid neural differentiable modeling framework designed for predicting spatiotemporal physics. This framework stands out due to its capability of quantifying associated uncertainty propagation in a scalable manner. At the core of our proposed method is the integration of the deep-ensemble based stochastic weight averaging Gaussian (SWAG) method with the unscented nonlinear transformation, devised to effectively estimate and propagate multi-source uncertainties inherent to hybrid neural models, offering both robust predictions and comprehensive uncertainty assessments. Specifically, our method discerns both aleatoric and epistemic uncertainties stemming from diverse sources, such as measurement noise and model form discrepancies, within a Bayesian model averaging framework. Aleatoric uncertainties, modelled under a Gaussian assumption, are propagated through the nonlinear components of the hybrid framework using the Unscented Transform and are captured by DNNs for unknown system components. On the other hand, epistemic uncertainties are estimated using the SWAG technique applied across an ensemble of models, which constructs an approximate posterior over both neural and physical parameters by leveraging multiple stochastic gradient descent (SGD) trajectories, each corresponding to a distinct local minimum encountered during training. A salient advantage of the proposed HybridNDiff-UQ is its straightforward implementation and amenability to parallelism. The design sidesteps extensive hyperparameter tuning, minimizing the chances of biased outcomes. Moreover, incorporating UQ into HybridNDiff provides several practical benefits. It yields full predictive distributions, allowing users to assess the reliability of predictions at each location. By expressing uncertainty in regions with sparse data or extrapolative behavior, it acts as a regularizer, improving robustness to overfitting and enhancing performance under distribution shifts. The ensemble-based Bayesian approach allows the model to capture multiple plausible solutions in ill-posed or multi-output problems, which deterministic models typically collapse into a single average. Additionally, epistemic uncertainty maps offer a principled way to identify where the model lacks confidence, enabling efficient active learning and iterative data acquisition strategies. This endeavor aims to fill the research gap concerning uncertainty propagation and quantification in physics-integrated differentiable hybrid neural models, bolstering the reliability and robustness of their predictions. The rest of the paper is organized as follows: Section 2 details the proposed methodology, including problem formulation from a Bayesian perspective and modeling of both aleatoric and epistemic uncertainties. The merits of the proposed model have been demonstrated in a variety of numerical experiments in Section 3. Finally, Section 4 concludes the paper.

## 2. Methodology

### 2.1. Problem formulation

In various scientific and engineering disciplines, partial differential equations (PDEs) are ubiquitously employed as a mathematical formalism to describe the behaviour of continua, including but not limited to fluid dynamics, solid mechanics, heat and mass transfer, electromagnetic fields, and quantum mechanical systems. However, for many complex systems, the analytical form of the governing PDEs is often partially known, which can be generally represented as

(1a) $$\frac{\partial v}{\partial t}=ℱ\left[𝒦\left(v\left(\overset{-}{x}\right);\lambda_{𝒦},\lambda_{𝒱}\right),𝒱\left(v\left(\overset{-}{x}\right);\lambda_{𝒦},\lambda_{𝒱}\right)\right],\;\overset{-}{x}\in \Omega_{p,t}$$

(1b) $$ℬ𝒞\left(v(\overset{-}{x});\lambda_{𝒦},\lambda_{𝒱}\right)=0,\;\overset{-}{x}\in \partial \Omega_{p,t}$$

(1c) $$ℐ𝒞\left(v\left(\overset{-}{x}\right);\lambda_{𝒦},\lambda_{𝒱}\right)=0,\;\overset{-}{x}\in \Omega_{p,t=0}$$

where $\bar{x}=\{x,t\}$ represents the spatiotemporal coordinates, with x specifically denoting spatial coordinates and t representing time. The spatiotemporal domain $\Omega_{p,t}$ is defined as the Cartesian product $\Omega_{p}\times [0,T_{r}]$, where $\Omega_{p}$ represents the spatial extent of the physical domain and $T_{r}$ denotes the time range under consideration; $\partial \Omega_{p,t}≜\partial \Omega_{p}\times [0,T_{r}]$ with $\partial \Omega_{p}$ representing the boundary of the physical domain. The nonlinear functions 𝒦(⋅) and 𝒱(⋅) characterize the known and unknown segments of the PDEs, respectively, which are coupled by a nonlinear functional ℱ(⋅). These functions are intrinsically reliant on certain physical parameters, which may sometimes remain elusive or uncertain. In this formulation, $\lambda_{𝒦}$ and $\lambda_{𝒱}$ denote the known and unknown physical parameters, respectively. Furthermore, the initial and boundary conditions can be abstractly represented by the differential operators ℐ𝒞 and ℬ𝒞, respectively, which may also be contingent upon specific parameters.

Direct physics-based modeling faces substantial challenges due to the incompleteness of the governing equation. Addressing this intricate issue, hybrid neural differential (HybridNDiff) models such as the PiNDiff model [13] have emerged to integrate neural networks with the known part of the PDEs using differentiable programming (DP) to learn the physics from data. Within this paradigm, unknown functions/operators such as ℱ(⋅) and 𝒱(⋅), are modeled as DNNs—${ℱ}_{nn}$ and ${𝒱}_{nn}$—with trainable parameters $\theta_{nn}$. Simultaneously, the elusive physical parameters $\lambda_{𝒱}$ are rendered trainable. A salient feature of the HybridNDiff models lies in their construction using DP, allowing the joint optimization of both DNNs and unknown physical parameters $\lambda_{𝒱}$ within a unified training environment through gradient back-propagation. The training of HybridNDiff model can be formulated as a PDE-constrained optimization problem, where the parameters to be optimized are collectively represented as $\theta ={\left[\theta_{nn},\lambda_{𝒱}\right]}^{T}\in R^{d}$. Given data $𝒟={\left\{{\bar{x}}_{i},v_{i}\right\}}_{i=1}^{N}$, these trainable parameters are optimized by minimizing the objective function, $J(v_{\theta }(\bar{x}),𝒟)$, defined, for example, by MSE or negative log-likelihood.

The optimization procedure is expressed as follows,

(2) $$\text{min}\;J(v_{\theta }(\bar{x}),𝒟;\theta )\;\text{s.t.}\;\frac{\partial v_{\theta }(x)}{\partial t}={ℱ}_{nn}[𝒦(v_{\theta }(\bar{x});\lambda_{𝒦},\lambda_{𝒱}),{𝒱}_{nn}(v_{\theta }(\bar{x});\lambda_{𝒦},\lambda_{𝒱},\theta_{nn});\theta_{nn}],\;\;\bar{x}\in \Omega_{p,t},\;ℬ𝒞(v_{\theta }(\bar{x});\lambda_{𝒦},\lambda_{𝒱})=0,\;\;\bar{x}\in \partial \Omega_{p,t},\;ℐ𝒞(v_{\theta }(\bar{x});\lambda_{𝒦},\lambda_{𝒱})=0,\;\;\bar{x}\in \Omega_{p,t=0}.$$

As a result of the end-to-end training process, the HybridNDiff model ensures that the solution vector $v_{\theta }(\bar{x})=HybridNDiff(\bar{x},\theta )$ consistently satisfies the governing PDEs for the given set of parameters θ. While HybridNDiff models, as described above, exhibit significant advantages over conventional black-box DNNs in predictive performance [13], they are not without vulnerabilities, especially when applied to out-of-training scenarios. This limitation raises questions about the reliability and robustness of the model’s predictions, making the estimation and propagation of uncertainty through hybrid neural solvers a crucial yet challenging endeavor. To this end, we propose the HybridNDiff-UQ framework, which employs an ensemble-based stochastic weight averaging Gaussian (En-SWAG) method to estimate the epistemic uncertainty and utilizes the unscented transformation technique for the propagation of Gaussian-based aleatoric uncertainty through the nonlinear functions. This approach aims to substantially improve the reliability and robustness of HybridNDiff models by offering not just accurate but also uncertainty-quantified predictions.

### 2.2. Overview of HybridNDiff-UQ framework

To effectively quantify the inherent uncertainties associated with the predicted state variable $v_{\theta }(\tilde{x})$, it is modeled as a random variable, denoted by $V(\tilde{x})$ conditioned on modeling parameters θ, where $\tilde{x}$ represents the inputs of the model including spatiotemporal coordinates $\tilde{x}$ and other nontrainable input parameters in the governing PDEs and IC/BCs. In this framework, the hybrid neural model is designed to yield the probability distribution $p(V|\tilde{x},\theta )$ for random variable V corresponding to a specified input $\tilde{x}$ and associated model parameters θ. Given a dataset 𝒟, the model can be trained following Bayesian principles, which is to determine the posterior distribution of the modeling parameters as p(θ∣𝒟). Namely, the output of the trained HybridNDiff-UQ model is the probability distribution $p(V|\tilde{x},𝒟)$ of the predicted states V, conditional on the training dataset 𝒟, which can be derived using Bayesian model averaging (BMA),

(3) $$p\left(V|\tilde{x},𝒟\right)=\int \;p\left(V|\tilde{x},\theta \right)p\left(\theta |𝒟\right)d\theta \;,$$

where posterior distribution p(θ∣𝒟) of trainable parameters is computed using Bayes’ theorem,

(4) $$p(\theta |𝒟)=\frac{p(𝒟|⨔)p(\theta )}{\int \;p(𝒟|\theta )p(\theta )d\theta },$$

where p(θ) is our prior beliefs about θ and p(𝒟∣⨔) is the joint likelihood of the datasest given the model prediction V with specific $\tilde{x}$ and θ. As the measurement noise of the dataset $𝒟={\left\{{\tilde{x}}_{i},V_{𝒟,i}\right\}}_{i=1}^{N}$ is usually independent and identically distributed, the joint likelihood can be expressed as $p(𝒟|⨔)=\prod_{i=1}^{N}p(v_{𝒟,i}|{\tilde{x}}_{i},\theta )$.

The integral in Eq. (3) can be estimated via Monte Carlo method in practice,

(5) $$p\left(V|\tilde{x},𝒟\right)\approx \frac{1}{M}\sum_{j=1}^{M}\;p\left(V|\tilde{x},\theta^{\left(j\right)}\right),\;\theta^{\left(j\right)}∼p\left(\theta |𝒟\right),$$

where ${\left\{\theta^{\left(j\right)}\right\}}_{j=1}^{M}$ are M Monte Carlo samples obtained from the posterior distribution p(θ∣𝒟). Through this approximation, the probabilistic prediction of $\left(V|\tilde{x},𝒟\right)$ essentially becomes a mixture of density functions conditioned on θ samples. Typically, one of the primary metrics to express uncertainty is the variance (often used to define the credible interval), which can be decomposed as follows using the law of total variance:

(6) $$V\mathrm{ar}\left(V|\tilde{x},𝒟\right)=\underset{Aleatoric\;}{\underbrace{E_{\theta |𝒟}\left(V\mathrm{ar}\left(V|\tilde{x},\theta \right)\right)}}+\underset{Epistemic\;}{\underbrace{{V\mathrm{ar}}_{\theta |𝒟}\left(E\left(V|\tilde{x},\theta \right)\right)}},\;$$

where the aleatoric uncertainty captures inherent system variabilities like measurement noise, while the epistemic uncertainty reflects the inadequacy of our model, stemming from incomplete physical understanding and the intricacies of neural network predictions. Up to now, two primary challenges still remain unresolved: (1) effectively modeling the likelihood function to capture the aleatoric uncertainty; (2) addressing the practical difficulties in sampling the posterior p(θ∣𝒟), given the extremely high dimensionality of the parameter space inherent to the hybrid neural model. Subsequent subsections will delve into our solutions for these challenges within the proposed HybridNDiff-UQ framework.

### 2.3. Modeling likelihood via HybridNDiff model for aleatoric UQ

To account for aleatoric uncertainty, the measurement noise ϵ of the state variables is assumed to be an additive Gaussian noise with zero mean,

(7) $$V|\tilde{x},\theta ={\overset{-}{v}}_{\theta }\left(\tilde{x}\right)+\epsilon_{\theta }$$

Namely, the conditional probability of the state $\left(V|\tilde{x},\theta \right)$ is Gaussian,

(8) $$p\left(V|\tilde{x},\theta \right)=𝒩\left({\overset{-}{v}}_{\theta }\left(\tilde{x}\right),\mathrm{diag}\left(\Sigma_{V,\theta }\right)\left(\tilde{x}\right)\right),$$

where the mean ${\bar{v}}_{\theta }(\tilde{x})$ and diagonal covariance matrix $\text{diag}(\Sigma_{V,\theta })(\tilde{x})$ are approximated by the differentiable hybrid neural model with trainable parameters θ. In other words, the output of the HybridNDiff solver is the probability distribution of the predicted state variable, characterized by it’s first and second moments,

(9) $${\overset{-}{v}}_{\theta }\left(\tilde{x}\right),\mathrm{diag}\left(\Sigma_{V,\theta }\right)\left(\tilde{x}\right)=HybridNDiff\left(\tilde{x};\theta \right).$$

Given the dataset $𝒟={\left\{{\tilde{x}}_{i},v_{𝒟,i}\right\}}_{i=1}^{N}$, the joint likelihood is computed as

(10) $$p(𝒟|\theta )=\prod_{i=1}^{N}\frac{1}{\sqrt{{\left(2\pi \right)}^{d}\text{det}\;(\Sigma_{V,\theta })}}\;\;\text{exp}\left\{-\frac{1}{2}{\left[v_{𝒟,i}-{\bar{v}}_{\theta }({\tilde{x}}_{i})\right]}^{T}\Sigma_{V,\theta }^{-1}({\tilde{x}}_{i})\left[v_{𝒟,i}-{\bar{v}}_{\theta }({\tilde{x}}_{i})\right]\right\},$$

where d is the dimension of the spatiotemporal state. When disregarding the uncertainty associated with θ, the training of the HybridNDiff model can be formulated as the minimization of the negative log-likelihood,

(11) $$\theta^{*}=\underset{\theta }{min}\;J\left(𝒟,\left(V|\tilde{x},\theta \right)\right)=\underset{\theta }{min}\;-\log p\left(𝒟|\theta \right).\;$$

This process allows the model to quantify the uncertainty arising from the noise present in the dataset 𝒟. However, a crucial point to bear in mind is that this training merely yields a single realization, i.e., a point estimate $\theta^{*}$, out of the full posterior distribution p(θ∣𝒟). Consequently, this model is unable to capture epistemic uncertainty that originates from HybridNDiff model parameterization. To truly gauge this, it becomes imperative to also approximate the parameter posterior and assess the total uncertainty as described in Eq. (5). Using Monte Carlo based BMA, the probability distribution of the prediction $p(V|\tilde{x},𝒟)$ given data becomes a Gaussian mixture, with its mean and variance (i.e., diagonal part of its covariance matrix) as,

(12a) $$E\left(V|\tilde{x},𝒟\right)\approx \frac{1}{M}\sum_{j=1}^{M}\;{\overset{-}{v}}_{\theta }\left(\tilde{x};\theta^{\left(j\right)}\right),\;$$

(12b) $$V\text{ar}\left(V|\tilde{x},𝒟\right)\approx \underset{\text{Aleatoric}}{\underset{︸}{\frac{1}{M}\sum_{j=1}^{M}\text{diag}(\Sigma_{V,\theta }(\tilde{x};\theta^{\left(j\right)}))}}\;\;+\underset{\text{Epistemic}}{\underset{︸}{\frac{1}{M}\sum_{j=1}^{M}{\left[{\bar{v}}_{\theta }(\tilde{x};\theta^{\left(j\right)})-E(V|\tilde{x},𝒟)\right]}^{2}}}.$$

Section 2.5 will delve into the practical methods for approximating the posterior p(θ∣𝒟).

### 2.4. Aleatoric uncertainty propagation within HybridNDiff framework

In this subsection, we elaborate on the design of the HybridNDiff framework for likelihood modeling. As indicated in Eq. (9), the output of the HybridNDiff model represents the probability distribution of the predicted state variable, which is primarily characterized by its mean and variance. This necessitates the formulation of a model adept at processing and predicting these statistical attributes (i.e., first- and second-order moments). As discussed in Section 2.1, the HybridNDiff framework is a implicit neural network defined by the partially known governing PDEs. Namely, the stochastic state $V_{\theta }(\bar{x})$ is modeled implicitly as,

(13a) $$\begin{array}{c} \frac{\partial V_{\theta }\left(\overset{-}{x}\right)}{\partial t}={ℱ}_{nn}\left[𝒦\left(V_{\theta }\left(\overset{-}{x}\right);\lambda_{𝒦},\lambda_{𝒱}\right),{𝒱}_{nn}\left(V_{\theta }\left(\overset{-}{x}\right);\lambda_{𝒦},\lambda_{𝒱},\theta_{nn}\right);\theta_{nn}\right]\\ \overset{-}{x}\in \Omega_{p,t} \end{array},$$

(13b) $$B𝒞\left(V_{\theta }(\overset{-}{x});\lambda_{𝒦},\lambda_{𝒱}\right)=0,\;\overset{-}{x}\in \partial \Omega_{p,t},$$

(13c) $$ℐ𝒞\left(V_{\theta }(\overset{-}{x});\lambda_{𝒦},\lambda_{𝒱},\Sigma_{0}^{2}\right)=0,\;\overset{-}{x}\in \Omega_{p,t=0}.$$

**Fig. 1.**
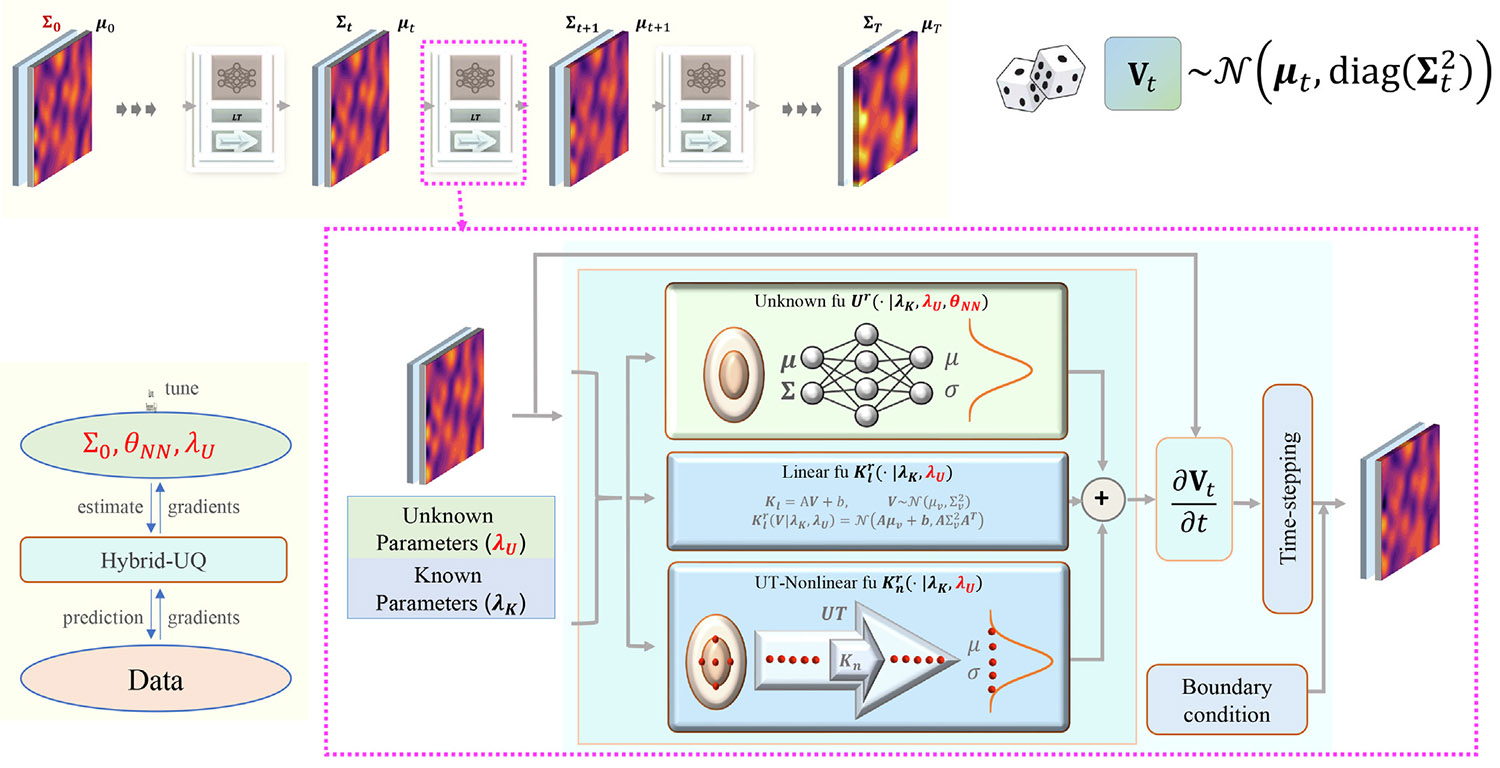
The overview of the auto-regressive HybridNDiff architecture for uncertainty propagation.

When formulating auto-regressive structure for temporal evolution, the architecture of the HybridNDiff neural for uncertainty propagation is depicted in Fig. 1.

In contrast to deterministic HybridNDiff models [12,13], the operators 𝒦, 𝒱, and ${ℱ}_{nn}$ here are designed to handle probabilistic distribution rather than deterministic values. To this end, all neural networks within this architecture are configured to process the mean and covariance at each grid point as inputs, producing dual outputs that represent the estimated mean and covariance of the intermediate functions. This design enables the HybridNDiff model to learn the spatiotemporal dynamics via the prediction mean ${\bar{v}}_{\theta }(\tilde{x})$, and also to quantify the associated aleatoric uncertainty via the diagonal covariance estimation, $\text{diag}\left(\Sigma_{V,\theta }^{2}(\bar{x})\right)$, which are spatiotemporal fields as well. The initial diagnoal covariance values are set as $\text{diag}\left(\Sigma_{V,\theta }^{2}(x,t=0)\right)=\theta_{\Sigma_{0}^{2}}$, which are trainable. These values will be propagated over time following the autoregressive temporal evolution, as illustrated in Fig. 1 . In the current model design, the $\text{diag}(\Sigma^{2})$ value at the boundary is assumed to be zero. The trainable parameters of the HybridNDiff-UQ model include DNN parameters, unknown physical coefficient, initial diagonal covariance matrix, collectively denoted as $\theta =(\theta_{\lambda_{U}},\theta_{𝒩𝒩},\theta_{\Sigma_{0}^{2}})$. Detailed DNNs architecture used for this study can be found in Appendix C2.

The PDE operators within the known segment of the right-hand side (RHS) of the governing equation (Eq. (13)) require discretization to formulate the predefined convolution layers—i.e., convolutional layers with fixed, non-trainable kernels that encode known physics, rather than learned filters. The kernel matrices are determined by the chosen discretization scheme. For example, when using the finite difference method to discretize spatial derivatives, the resulting finite difference stencil can be directly translated into a convolutional kernel. Consider a 2D Laplacian operator which in central difference form is approximated as $\nabla^{2}v\approx \frac{v_{i-1}-2v_{i}+v_{i+1}}{\Delta x^{2}}+\frac{v_{j-1}-2v_{j}+v_{j+1}}{\Delta y^{2}}$. The five-point stencil for the Laplacian operator $\nabla^{2}v\;$ can be directly encoded as a 3 × 3 convolutional kernel. Similarly, other differential operators can be directly encoded as convolutional kernels [11]. Detailed information on the discretization process is available in Appendix B. After discretization, these discretized known operators need to manage the propagation of uncertainty on the grid level. We consider two types of operators in this context: linear and nonlinear known PDE operators. For linear operators ${𝒦}_{l}$, such as discretized diffusion terms $\nabla^{2}$, the propagation of mean and covariance matrix of a random variable V is straightforward. This is because the affine transformation of a Gaussian distribution result in another Gaussian distribution. Specifically, we have

(14) $${𝒦}_{l}\left(𝒩\left({\overset{-}{v}}_{\theta },\Sigma_{V,\theta }^{2}\right)\right)=𝒩\left(K{\overset{-}{v}}_{\theta }+b,K\Sigma_{V,\theta }^{2}K^{T}\right),\;$$

where the linear PDE operator is defined as ${𝒦}_{l}(v)=Kv+b$.

**Fig. 2.**
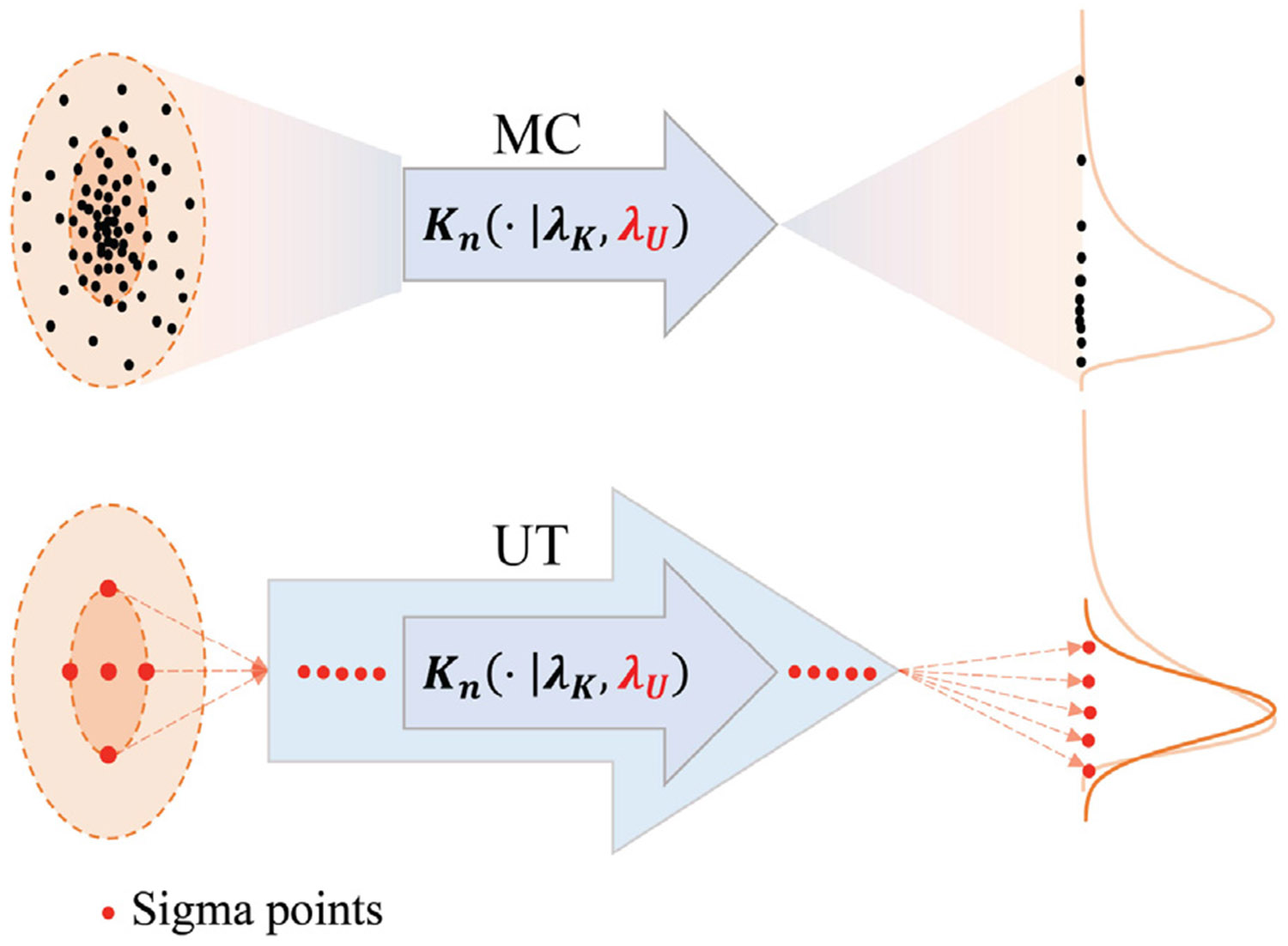
Illustrative plot of unscented transformation (UT) versus Monte Carlo (MC) methods for uncertainty propagation.

When it comes to noninear PDE operators, ${𝒦}_{n}$, the propagation of mean and variance is more challenging, as it typically requires either linearization of the operator, which often compromises accuracy, or the use of Monte Carlo sampling methods, which can be computationally intensive if not infeasible. To address these challenges, our work utilizes the unscented transform method [57]. The unscented transform (UT) is a mathematical technique used for filtering and smoothing in nonlinear dynamical systems. It aims to accurately propagate the first and second moments of a probability distribution through nonlinear mappings. The method involves generating a small, deterministically selected set of representative samples, known as sigma points, which are designed to effectively capture the mean and covariance of the input distribution. Unlike traditional Monte Carlo sampling, which relies on a large number of random samples, the UT strategically selects 2L+1 sigma points (where L is the dimensionality of the input random variable) around the mean of a distribution. These points are symmetrically placed and weighted such that their sample mean and covariance match the true distribution up to second order. When propagated through a nonlinear function, the resulting transformed sigma points are used to compute an accurate estimate of the mean and covariance of the output distribution. (More implementation details on UT can be found in Appendix C1) The number of sigma points used is small, greatly enhancing efficiency over Monte Carlo methods. After undergoing the nonlinear transformation, the mean and covariance of the new distribution are recalculated from these transformed sigma points through a weighted summation, with weights as specified in [57]. This method is particularly advantageous because it avoids the need to compute Jacobians, as required in linearization-based approaches like the Extended Kalman Filter. It also offers improved accuracy in highly nonlinear settings or when accurately capturing the tails of the distribution is important [57]. An illustrative plot of the UT vs. MC-based uncertainty propagation is shown in Fig. 2.

### 2.5. Scalable posterior sampling for epistemic UQ

**Fig. 3.**
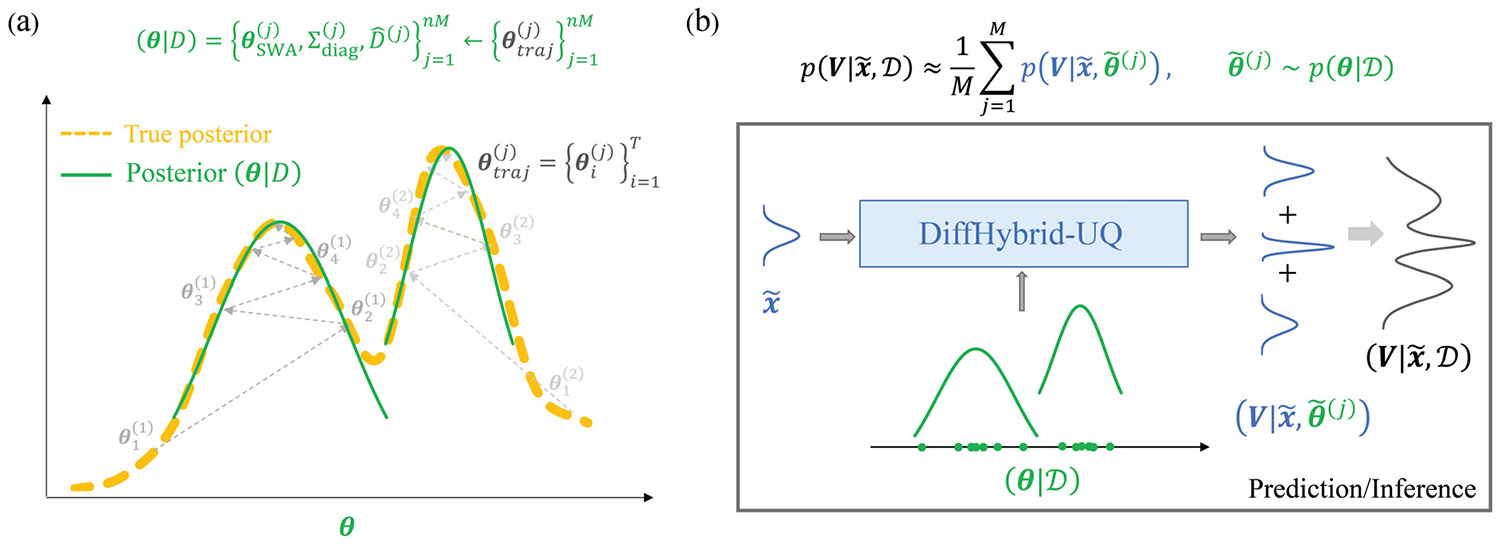
(a) Schematic of the estimation of the posterior distribution using the ensemble-based SWAG training. (b) Prediction/Inference using the posterior distribution.

Direct computation of the posterior p(θ∣𝒟) using traditional Bayesian sampling techniques, e.g., MCMC, is impractical for deep learning models due to their high dimensionality, which slows convergence and requires extensive computation. Additionally, DNNs often present complex posterior landscapes that these methods struggle to navigate, leading to memory constraints and convergence issues. To overcome these limitations, ensemble-based techniques have emerged as a promising alternative, offering scalable and efficient approaches to capture epistemic uncertainty. In particular, the ensemble strategy starts with different initial samples and applies stochastic gradient descent to locate a local maximum of the posterior distribution, each corresponding to a different mode, thereby providing a more robust representation of model-form uncertainty [38]. In this study, we employ the Stochastic weight averaging Gaussian (SWAG) [55], in combination with a DeepEnsemble framework [53], to approximate the posterior p(θ∣𝒟). This approach is grounded in the hypothesis by Mandt et al. [58], suggesting that stochastic gradient descent (SGD) iterations can empirically approximate a Markov chain of samples with a stationary distribution. Here, an SGD trajectory is interpreted as an approximate Bayesian MC sampler, effectively estimating the local geometry of the posterior distribution of the trainable parameters θ. To enhance scalability and simplify the process, the local posterior geometry is characterized primarily by its first and second moments, with the low-rank covariance approximated. This involves collecting only the running time mean and variance of θ along the SGD trajectory to form a Gaussian approximation of the local posterior, a method known as SWAG. Given the complex and multimodal nature of DNN posterior landscapes, SWAG is ideally integrated within the DeepEnsemble framework. As illustrated in Fig. 3, this approach entails independently obtaining an ensemble of SGD trajectories in parallel, with each trajectory potentially approximating different peaks of the posterior. This method not only simplifies the representation of the posterior but also ensures a comprehensive exploration of the model’s parameter space, significantly enhancing the accuracy and reliability of epistemic uncertainty quantification in hybrid neural models.

Specifically, the training involves multiple $\left(N_{m}\right)$ instances of the HybridNDiff model, each initialized with a distinct set of initial parameters $\theta_{0}^{\left(k\right)},k=1,2,\ldots ,N_{m}$. Prior studies suggest that ensembles of size 510 effectively capture epistemic uncertainty while maintaining reasonable computational cost. Consistent with this, our experiments showed diminishing returns beyond 10 models. Therefore, we use $N_{m}=10$ in this study. These models are independently updated using the SWAG algorithm. For each SGD trajectory $\theta_{\text{traj}}^{\left(k\right)}={\left\{\theta_{i}^{\left(k\right)}\right\}}_{i=1}^{T}$, the Gaussian statistics $\left\{\theta_{\text{SWA}}^{\left(k\right)},\Sigma_{\text{diag}}^{\left(k\right)},{\hat{D}}^{\left(k\right)}\right\}$ are computed on the fly. Here $\theta_{\text{SWA}}^{\left(k\right)}$ is the mean, $\Sigma_{\text{diag}}^{\left(k\right)}$ is diagonal covariance matrix, and ${\hat{D}}^{\left(k\right)}=\theta_{\text{traj}}^{\left(k\right)}-\theta_{\text{SWA}}^{\left(k\right)}$ is the deviation matrix for given $\theta_{\text{traj}}^{\left(k\right)}$, respectively. To limit the rank and reduce the memory usage, ${\hat{D}}^{\left(k\right)}$ is often estimated by retaining only the last r vectors of ${\hat{D}}^{\left(k\right)}$ corresponding to the last r samples obtained during training, thereby the deviation matrix results in ${\hat{D}}^{\left(k\right)}=[d_{T-r+1}^{\left(k\right)},d_{T-r+2}^{\left(k\right)},\ldots ,d_{T}^{\left(k\right)}]$ with column vector $d_{k}^{\left(k\right)}=\theta_{k}^{\left(k\right)}-\theta_{\text{SWA}}^{\left(k\right)}$. Here T represents the total number of samples in each SGD trajectory. Notably, the sampling can be conducted intermittently, every few epochs, with the interval being adjustable. Consequently, the trajectory length T can be less than the total number of training epochs. Additionally, to maintain memory efficiency, only the key statistics, rather than the entire SGD trajectory $\theta_{\text{traj}}^{\left(k\right)}$, are stored during training. These treatments ensure the scalability and computational feasibility for large DL and hybrid neural models. Upon the completion of SWAG training, the local geometry of the posterior p(θ∣𝒟) is approximated by the Gaussian density defined by each set of $\left\{\theta_{\text{SWA}}^{\left(k\right)},\Sigma_{\text{diag}}^{\left(k\right)},{\hat{D}}^{\left(k\right)}\right\}$. Using the idea of the DeepEnsemble method, the full posterior distribution can be approximated by aggregating local Gaussian approximations obtained from multiple independent SWAG-trained models, where the full posterior can be sampled using the following equation,

(15a) $$\theta =\sum_{k=1}^{N_{m}}\;\delta \left(k,k^{'}\right){\tilde{\theta }}^{\left(k\right)},\;k^{'}∼𝒱\left\{0,1,2,\ldots ,N_{m}\right\},$$

(15b) where,δ(k,k′)={1,ifk=k′,0,otherwise,}

(15c) $$\begin{array}{rr} {\tilde{\theta }}^{\left(k\right)}=\theta_{SWA}^{\left(k\right)}+\frac{1}{\sqrt{2}}\cdot \Sigma_{diag}^{\left(k\right)}z_{1}+\frac{1}{\sqrt{2(r-1)}}{\overset{ˆ}{D}}^{\left(k\right)}z_{2}, & z_{1}∼𝒩\left(0,I_{d}\right),\\ & z_{2}∼𝒩\left(0,I_{r}\right),\; \end{array}$$

where d represents the number of training parameters in $\theta^{\left(k\right)}$; $I_{d}$ and $I_{r}$ are two identity matrices of ranks d and r, respectively. Using these sampled posterior realizations, the predictive mean along with the associated aleatoric and epistemic uncertainties can be computed via Eq. (12). SWAG effectively captures the posterior geometry around local minima, while the ensemble approach addresses the multimodal nature of the posterior, thereby facilitating the efficient estimation of epistemic/model uncertainty. Consequently, our approach involves training multiple HybridNDiff model instances with SWAG simultaneously to capture different Gaussian posterior distributions characterized by low-rank covariance matrices. The overall training algorithm of the proposed HybridNDiff-UQ model is detailed in Algorithm 1, and the SWAG implementation details are provided in Appendix A.

While the current HybridNDiff-UQ framework models uncertainty primarily through the first and second moments (mean and covariance), its architecture implicitly supports more expressive, non-Gaussian posterior approximations, achieved by combining deep ensembles with SWAG. Each ensemble member captures a distinct local mode of the posterior, and the local posterior around each mode is then approximated by a Gaussian using SWAG, thereby collectively forming a mixture-like approximation capable of capturing multi-modal and non-Gaussian characteristics. Although explicit propagation of higher-order moments (e.g., skewness, kurtosis) using techniques like higher-order Unscented Transforms could enhance fidelity, such methods increase computational complexity and assume unimodal Gaussian priors, limiting their flexibility in capturing sharp transitions or multi-modal structures.

## 3. Results

This section presents extensive numerical experiments conducted to assess the performance of the proposed HybridNDiff-UQ framework in modeling various dynamical systems governed by ODEs and PDEs, with a focus on uncertainty quantification.

**Table:** **Algorithm 1.** Training algorithm for HybridNDiff-UQ model.

Data:Read experimental data𝒟={xi,vi}k=1NInitialize:Initialize trainable parameters{θ0(k)}k=1Nm={θ0(1),θ0(2),…,θ0(Nm)}fori=1toN_epochdo∣▷Training multiple models with differentθ(k)forθi(k)={θi(k)}k=1Nmdo∣(vθ,ΣV,θ2)←HybridNDiff−UQ(Vinit(v0,θi,Σ02(k))),θi(k)=(θi,λU(k),θi,𝒩𝒩(k),θi,Σ02(k)))(vθ(p),ΣV,θ2(p))←Map_to_Observable(vθ,ΣV,θ2)▷Exp observablesℒ←log(ΣV,θ2(p))2+(Vi−vθ(p))22ΣV,θ2(p)▷Loss functionθi+1(k)←θi(k)−λ∇θ)ℒ(θi(k))▷Updateθ(k)parametersifMOD(i,interval)=0then∣n←i∕intervalθ¯(k)←nθ¯(k)+θi+1(k)n+1θ¯2(k)←nθ¯2(k)+θi+12(k)n+1▷Update movementsD^(k)←[di−r(k),…,di+1(k)]▷store devation∣end∣end∣end

### 3.1. Modeling dynamical systems governed by ODEs

We first study the proposed HybridNDiff-UQ model on the Hamiltonian systems governed by the simple ODEs described as follows,

(16a) $$\frac{\partial x_{1}}{\partial t}=f_{1}\left(x_{1},x_{2}\right)=x_{2},$$

(16b) $$\frac{\partial x_{2}}{\partial t}=f_{2}\left(x_{1},x_{2}\right)=-\sin\left(x_{1}\right),\;$$

where $v={\left[x_{1},x_{2}\right]}^{T}\in R^{2}$ is a two-dimensional state variable, and the functions $f_{1},f_{2}\;:R^{2}\Rightarrow R^{1}$ can be linear or nonlinear, defining the right-hand side of the governing equations. Synthetic data are generated by solving Eq. (16), and Gaussian random noise is added to simulate measurement uncertainties typical of real-world experimental data. To illustrate the HybridNDiff-UQ model’s capabilities, the governing physics is assumed to be only partially known. Specifically, while the function $f_{2}$ is known, $f_{1}$ is considered unknown. Here, $f_{2}$ is considered known to test the UT-based uncertainty propagation through non-linear function $f_{2}$. As a supplementary case, a scenario where $f_{1}$ is known and $f_{2}$ is considered unknown is also presented in Appendix E1. We construct an auto-regressive hybrid neural model based on Eq. (16), where a trainable Bayesian neural network (BNN), ${𝒱}_{nn}\;:R^{4}\Rightarrow R^{2}$ represents the right-hand side of Eq. (16a), processing the mean and variance of v to predict the Gaussian statistics of $f_{1}$. The known function $f_{2}$ is encapsulated within a UT function, resulting in a probabilistic function, $𝒦=UT(f_{2})\;:R^{4}\Rightarrow R^{2}$. Both ${𝒱}_{nn}$ and 𝒦 function as probabilistic entities, handling random variables to capture the inherent uncertainties in the training data.

During the training phase, the HybridNDiff-UQ model is trained with data spanning a single trajectory over the time interval $\left[0\;:\Delta t\;:T^{\prime }\right]$, where $T^{\prime }<T$. The model’s predictive performance is assessed in the forecast/testing range $\left(T^{\prime }\;:\Delta t\;:T\right]$. To reflect realistic experimental conditions, different measurement errors with variances of 0.5^2^ for $x_{1}$ and 0.2^2^ for $x_{2}$ are introduced, acknowledging that measurement uncertainty can differ across variables. To intensively assess the model’s capacity for uncertainty prediction, we conduct four test cases as enlisted in Table 1. These cases serve to test the model’s efficacy in accurately estimating uncertainties under varying data availability and measurement error conditions. In-depth analyses and results of these test cases will be discussed in subsequent sections.

#### 3.1.1. Case 1: Complete data for both state variables

**Fig. 4.**
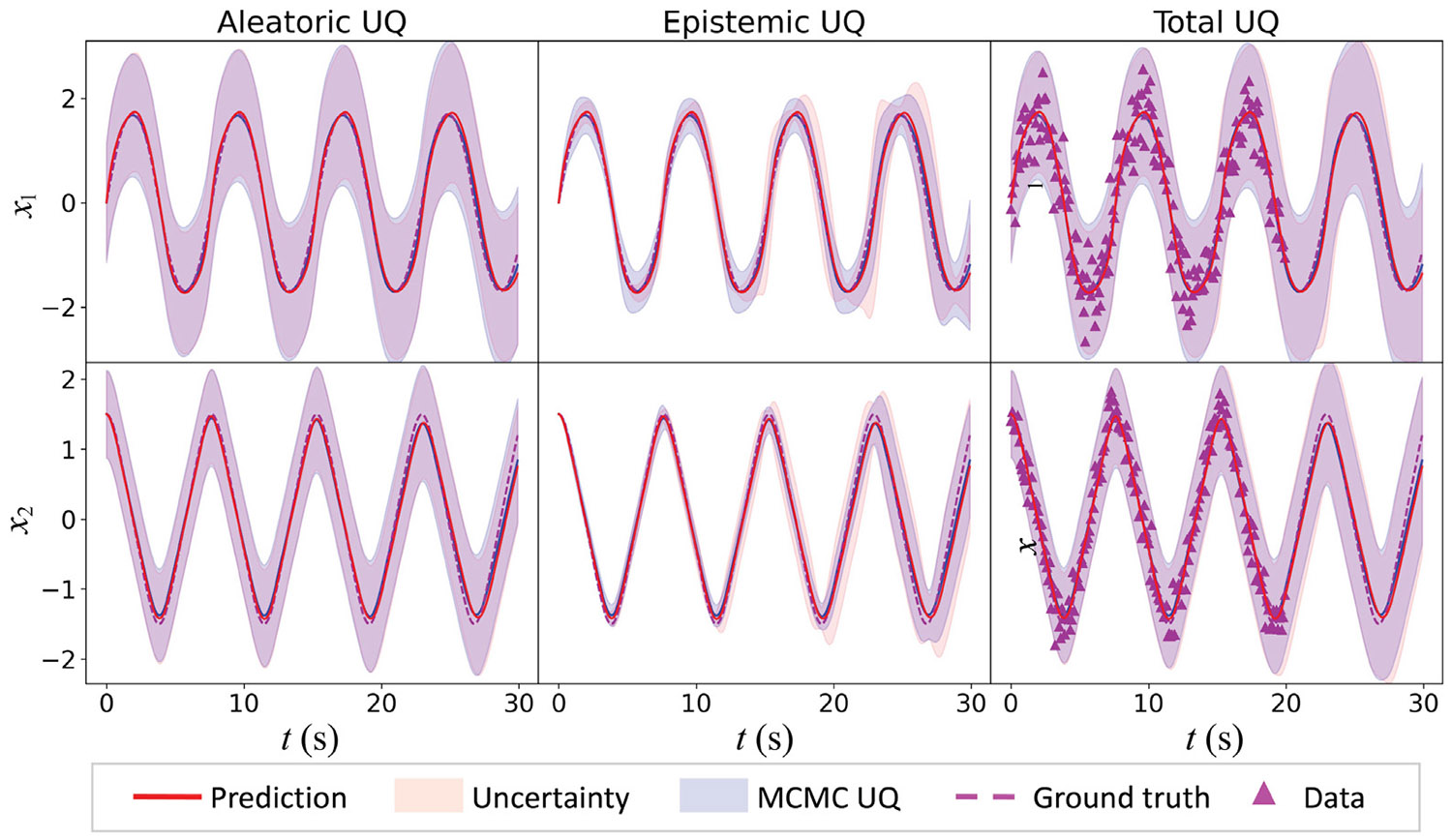
Compares the HybridNDiff-UQ model’s prediction and UQ against HMC method. Training data spans $𝒟=(x_{1},x_{2}\in {\left(R\right)}^{1}\times [0:0.1:20\;s])$ with testing in the $\left(x_{1},x_{2}\in {\left(R\right)}^{1}\times (20:0.1:30\;s]\right)$ region.

The HybridNDiff-UQ model is trained using data for both the $x_{1}$ and $x_{2}$ variables over 20 s interval $𝒟=(x_{1},x_{2}\in {\left(R\right)}^{1}\times [0\;:0.1\;:20\;s])$. The training, spanning 10,000 epochs, yields results depicted in Fig. 4. To verify the UQ performance of the proposed HybridNDiff-UQ model, we also perform exact Bayesian learning using the Hamiltonian Monte Carlo (HMC) method, often considered the gold standard for Bayesian UQ. For HMC, 100,000 samples are obtained, initialized at the local minima obtained from gradient descent training, thereby bypassing the burn-in phase to enhance computational efficiency. Notably, generating these samples required approximately over 24 h, even after extensive hyperparameter tuning, reflecting the high cost of HMC posterior sampling. HMC sampling does not scale well to complex, high-dimensional models. A side-by-side comparison of the HybridNDiff-UQ and HMC predictions is shown in Fig. 4, with color-shaded regions representing 3-STD confidence intervals.

The prediction mean of the HybridNDiff model aligns very well with the ground truth for both $x_{1}$ and $x_{2}$, even in the forecasting region (20 : 0.1 : 30 s], demonstrating the model’s forecasting capability. Overall, the total prediction uncertainty of the DiffHbrid-UQ model matches that of the HMC method. Notably, the shaded areas in the first column, indicative of aleatoric uncertainty, differ for variables $x_{1}$ and $x_{2}$, corresponding to the varying noise levels in the data, as shown in the third column. The aleatoric uncertainty estimated by the HybridNDiff-UQ model closely agrees with that of the HMC method, reflecting its effectiveness in capturing inherent data uncertainty. Regarding epistemic uncertainty, represented by shaded areas in the second column, these remain small but increase in the forecast region where data are unavailable. This rise in epistemic uncertainty is expected and reasonable due to the lack of data in this region. The epistemic uncertainty estimated by the HybridNDiff-UQ model closely aligns with that of the HMC method, demonstrating its effectiveness in exploring the multimodal posterior. Notably, the HybridNDiff-UQ model, trained for just 5 min over 10,000 epochs, captured uncertainty with an accuracy comparable to that of HMC, which required 100,000 samples and took approximately one day of computation. Similar results were found for the supplementary case with $f_{1}$ as known and $f_{2}$ unknown as presented in Appendix E1.

**Fig. 5.**
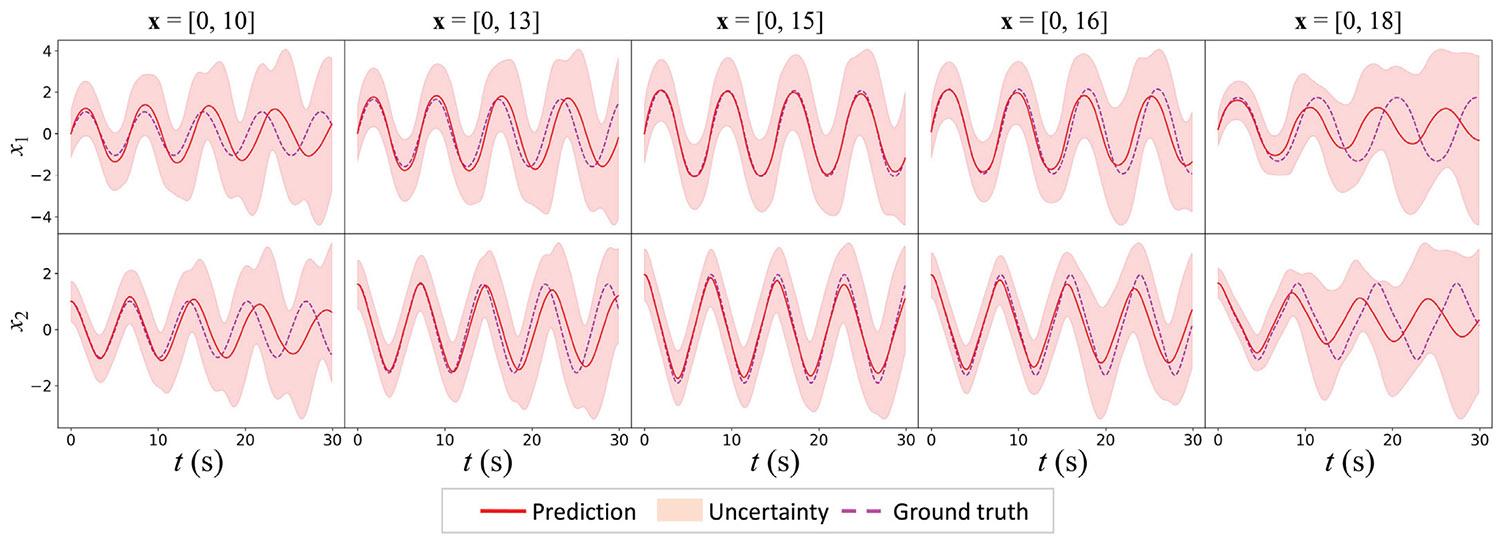
HybridNDiff-UQ prediction with quantified uncertainties compared against the ground truth for different testing initial conditions. Trained initial condition: x=[0,15].

To evaluate the generalization capability of the HybridNDiff learning framework, the model trained on one initial condition x=[0,15] is tested on various trajectories generated under different initial conditions. Fig. 5 shows how the model predictions compared with the ground truth across these scenarios. As predictions deviate from the training trajectory, initialized at x=[0,15], there is a notable decrease in mean prediction accuracy, accompanied by increased uncertainty. This trend, evident in the parametric extrapolation region, indicates the HybridNDiff-UQ model is able to reasonably estimate the confidence of its prediction, which diminish as it moves beyond the range of the training scenarios.

Overall, the HybridNDiff-UQ model exhibit promising performance, producing uncertainty estimates that are in line with those obtained from the HMC samping. Its ability to quantify both aleatoric and epistemic uncertainties offers valuable insights into the reliability of its predictions, effectively addressing the inherent uncertainties stemming from both data variability and model formulation.

#### 3.1.2. Case 2: Sparse data for both variables

**Fig. 6.**
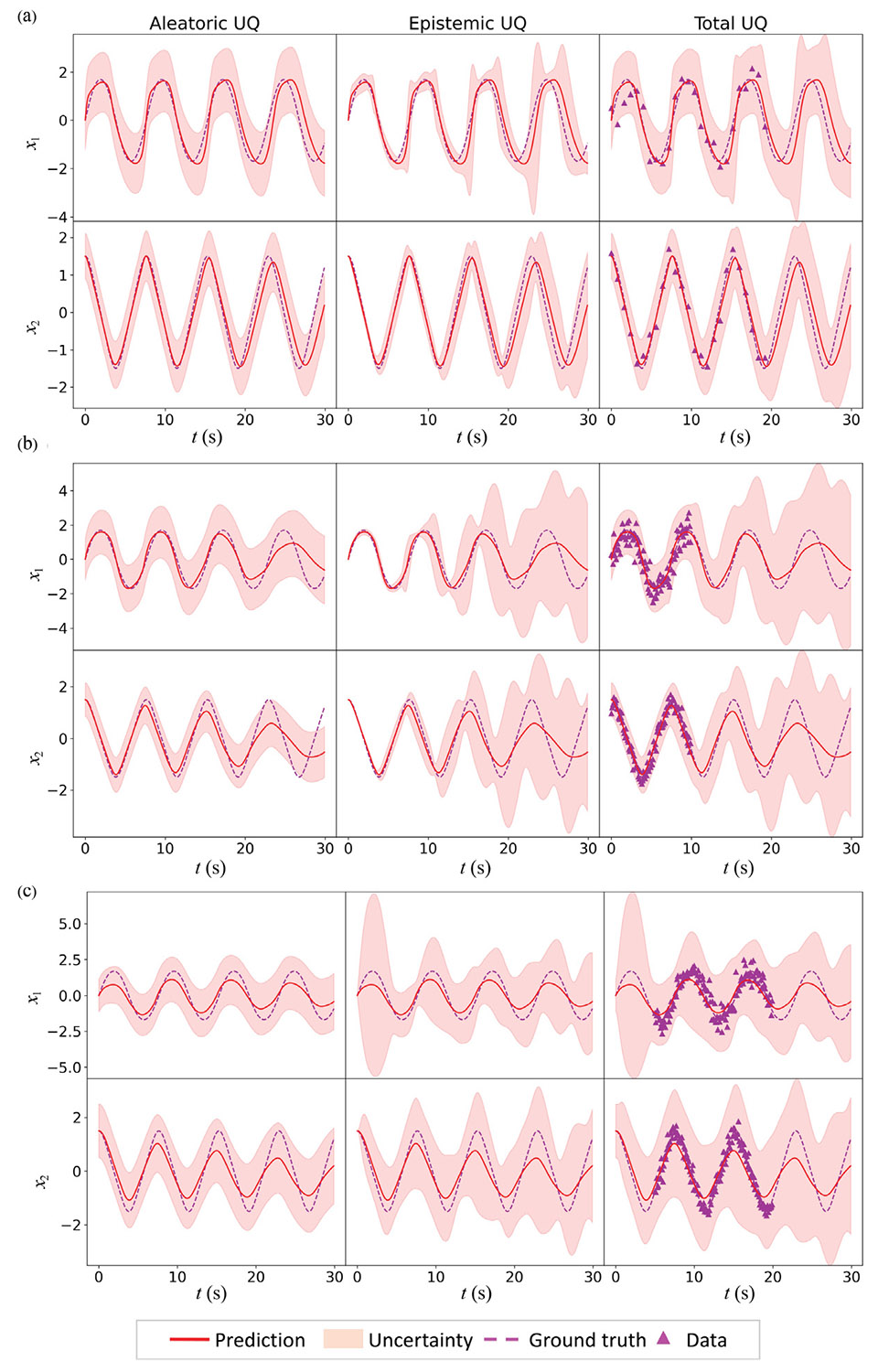
Comparison of the HybridNDiff-UQ model’s prediction with UQ against the ground truth. (a) Training data is $𝒟=(x_{1},x_{2}\in {\left(R\right)}^{1}\times [0:1:20\;s])$, with testing in $\left(x_{1},x_{2}\in {\left(R\right)}^{1}\times (20:0.1:30\;s]\right)$. (b) Training data is $𝒟=(x_{1},x_{2}\in {\left(R\right)}^{1}\times [0:0.1:10\;s])$, with testing in $\left(x_{1},x_{2}\in {\left(R\right)}^{1}\times (10:0.1:30\;s]\right)$. (c) Training data is $𝒟=(x_{1},x_{2}\in {\left(R\right)}^{1}\times [5:0.1:20\;s])$, with testing in $\left(x_{1},x_{2}\in {\left(R\right)}^{1}\times [0:0.1:5\;s)\cup (20:0.1:30\;s]\right)$.

In this scenario, the HybridNDiff-UQ model is trained with partial data for both the $x_{1}$ and $x_{2}$ variables. Specifically, three testing scenarios were considered. In the first scenario, the HybridNDiff-UQ model was trained on sparse time data of [0 : 1 : 20 s], denoted as $𝒟=(x_{1},x_{2}\in {\left(R\right)}^{1}\times [0\;:1\;:20\;s])$ for both $x_{1}$ and $x_{2}$. In the other two scenarios, the model was trained on data with a limited time range: one with a time range of [0 : 0.1 : 10 s], denoted as $𝒟=(x_{1},x_{2}\in {\left(R\right)}^{1}\times [0\;:0.1\;:10\;s])$, and the other with a time range of [5 : 0.1 : 20 s], denoted as $𝒟=(x_{1},x_{2}\in {\left(R\right)}^{1}\times [5\;:0.1\;:20\;s])$. Notably, in the third scenario, data for the initial 5 s is not included to evaluate the model’s performance in the presence of missing data. The model undergoes a training period of 10,000 epochs, with results presented in Fig. 6.

In the first scenario, the captured data uncertainty is lower than the inherent uncertainty present in the provided data due to the sparse data failing to adequately reflect the data uncertainty. The predicted epistemic uncertainty is higher in this scenario compared to the previous case 1, indicating the model’s lower confidence in predictions due to sparse training data. Despite this, the mean predictions remain reasonably accurate. For the second and the third scenarios, the confidence intervals for data uncertainty captured align well with the data uncertainty present in the provided data. However, the predicted epistemic uncertainty differs between these scenarios. In the second scenario, both $x_{1}$ and $x_{2}$ predictions deviate from the ground truth in the forecasting region (10 : 0.1 : 30 s], as the training data is limited to the short time range [0 : 0.1 : 10 s]. This is reflected by the increasing confidence interval in the forecasting region. In the third scenario, the prediction for $x_{1}$ shows a notable deviation from the ground truth, the model aptly indicates lower confidence in this region. Conversely, for $x_{2}$, the model’s uncertainty remains relatively low in the same interval, suggesting a good alignment between the HybridNDiff-UQ predictions and the ground truth. Overall, the model performs reasonably well in unseen data regions, with an expected increase in uncertainty, especially as it moves into the forecasting region. Thus, the HybridNDiff-UQ model showcases its ability to estimate uncertainties effectively, even with partially available data. By precisely capturing data uncertainties and indicating reduced confidence in data-sparse regions, the model offers valuable insights into the reliability of its predictions.

#### 3.1.3. Case 3: Incomplete data for only one state variable

**Fig. 7.**
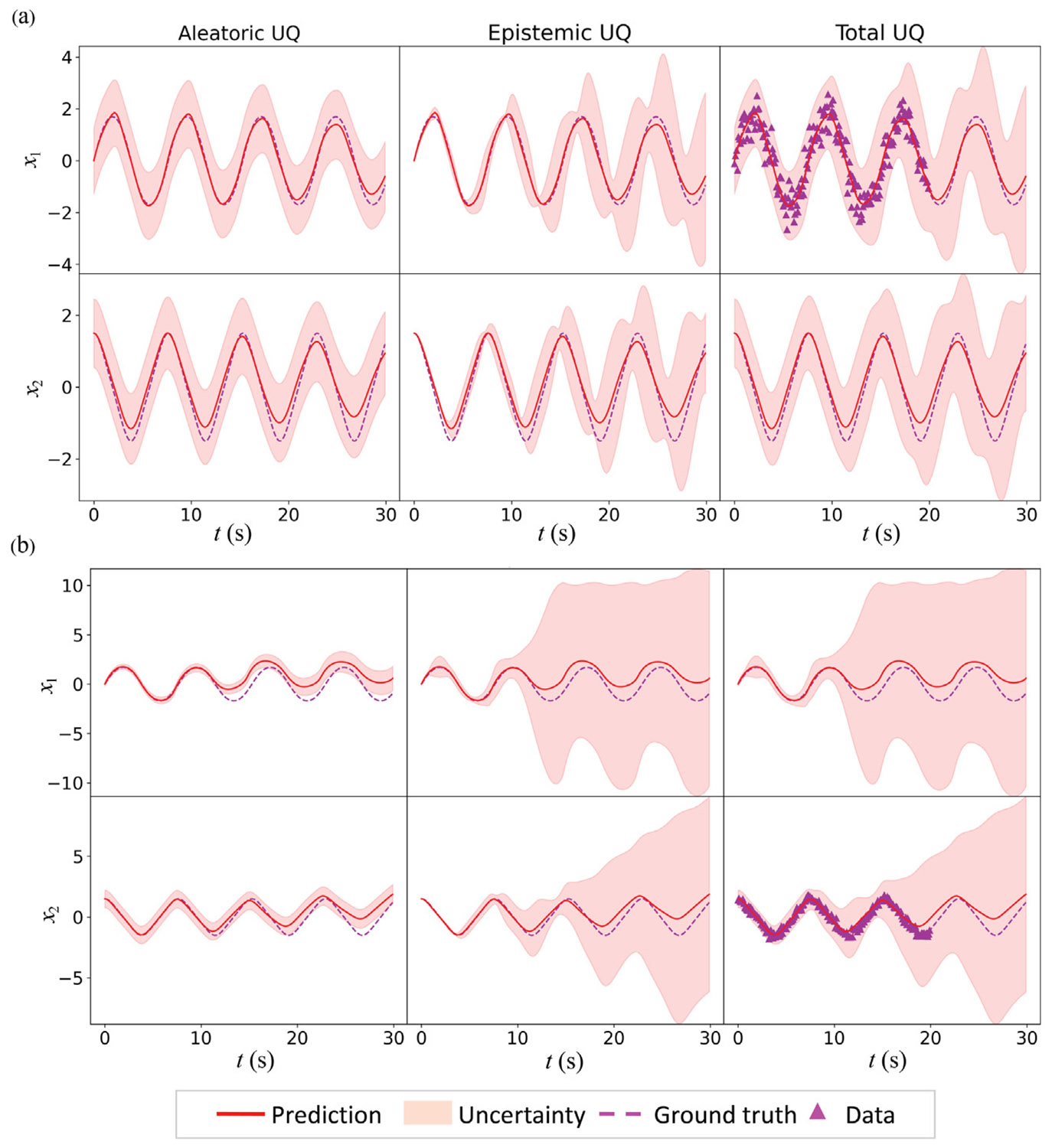
Comparison of the HybridNDiff-UQ model’s prediction with UQ against the ground truth. (a) Training data is $𝒟=(x_{1}\in {\left(R\right)}^{1}\times [0:0.1:20\;s])$, with testing in $\left(x_{1}\in {\left(R\right)}^{1}\times (20:0.1:30\;s],x_{2}\in {\left(R\right)}^{1}\times [0:0.1:30\;s]\right)$. (b) Training data is $𝒟=(x_{2}\in {\left(R\right)}^{1}\times [0:0.1:20\;s])$, with testing in $\left(x_{1}\in {\left(R\right)}^{1}\times [0:0.1:30\;s],x_{2}\in {\left(R\right)}^{1}\times (20:0.1:30\;s]\right)$.

A key strength of hybrid neural models lies in their ability to be trained with incomplete data, even when labels for certain state variables are absent. To demonstrate this capability, the HybridNDiff-UQ model was trained for two scenarios where the training data for only one of the $x_{1}$ and $x_{2}$ variables were provided. These datasets are designated as $𝒟=(x_{1}\in {\left(R\right)}^{1}\times [0:0.1:20\;s])$ and $𝒟=(x_{2}\in {\left(R\right)}^{1}\times [0:0.1:20\;s])$. This training, encompassing 10,000 epochs, produced results illustrated in Fig. 7.

The results indicate that when trained with $x_{1}$ data, the mean predictions are accurate, and this accuracy is accordingly reflected in confidence interval for both $x_{1}$ and $x_{2}$. This precise prediction can be attributed to the fact that $f_{1}$ is being learned by NN, and the NN’s predictions directly influence the predictions for $x_{1}$, the variable for which the data is provided. Conversely, in the scenario where the training data for $x_{2}$ is provided, the results show that the HybridNDiff-UQ model effectively captures the dynamics of $x_{2}$, while the $x_{1}$ prediction mean notably deviates from the ground truth. This is due to that the information for $f_{1}$ is learned indirectly from the labeled data of $x_{2}$. For $x_{1}$, the model understandably exhibits much higher uncertainty as no direct labels are provided. In contrast, for $x_{2}$, the model displays relatively low uncertainty. In line with previous findings, confidence interval increases in the forecast region, reflecting the increased model uncertainty beyond the training data. Its ability to reflect data scarcity and increase uncertainty in unobserved regions highlights its robustness in limited data scenarios.

#### 3.1.4. Case 4: Effect of measurement error

**Fig. 8.**
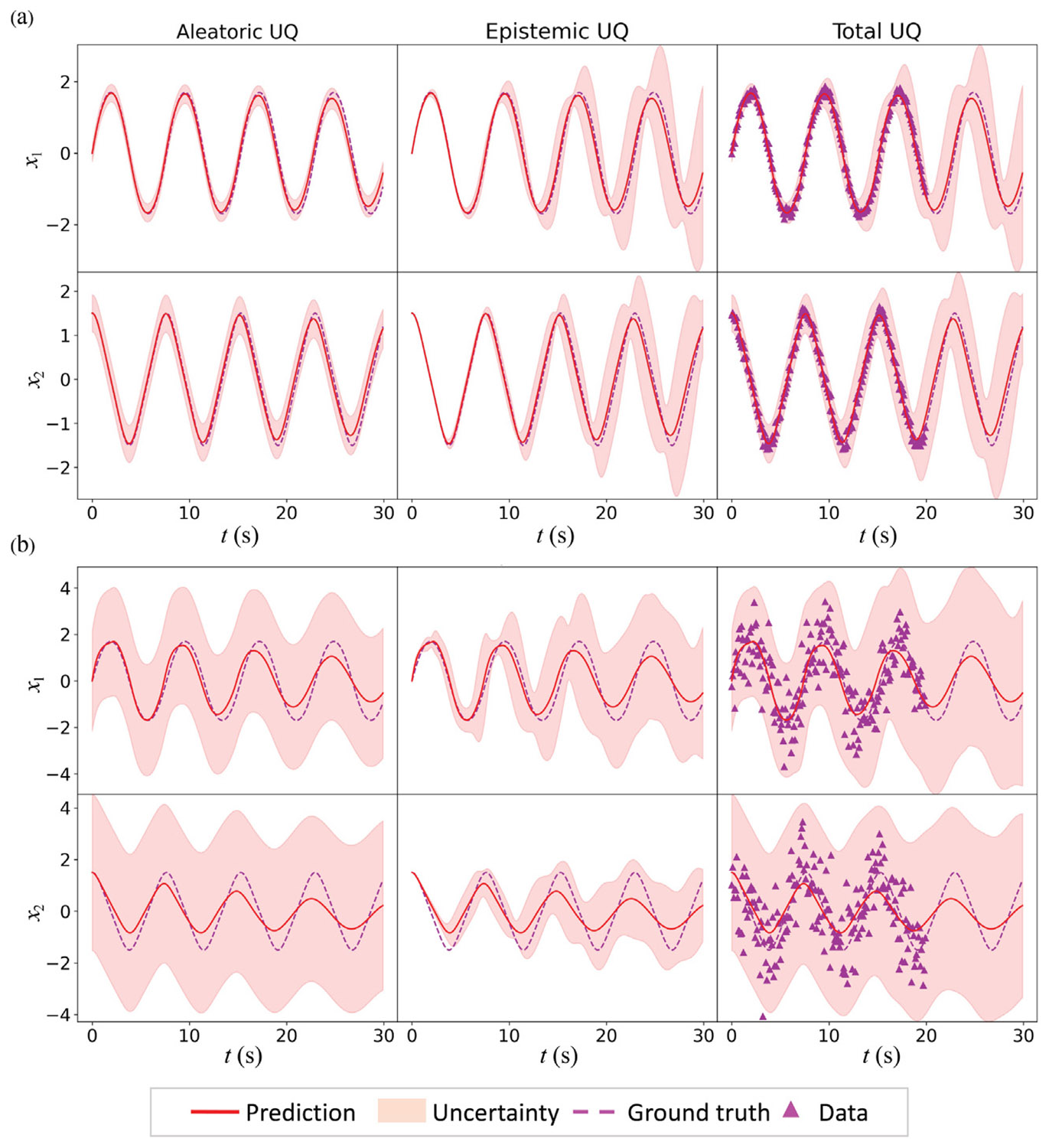
Comparison of the HybridNDiff-UQ model’s prediction with UQ against the ground truth for 0.1^2^ (a) and 1.0^2^ (b) measurement noise variance. Training data is $𝒟=(x_{1},x_{2}\in {\left(R\right)}^{1}\times [0:0.1:20\;s])$, with testing in $\left(x_{1},x_{2}\in {\left(R\right)}^{1}\times (20:0.1:30\;s]\right)$.

Finally, the variance of the measurement noise is varied to test the HybridNDiff-UQ model’s capability to infer the data uncertainty. Two scenarios were considered, one with 0.1^2^ and the other with 1.0^2^ variance in measurement noise. As can be seen in Fig. 8, the confidence interval for the data uncertainty prediction encloses all the data points for different measurement errors. Additionally, the epistemic uncertainty predicts lower confidence for the data with larger measurement noise, reflecting the HybridNDiff-UQ model’s ability to quantify the uncertainty for the inferred dynamics from noisy data.

### 3.2. Modeling spatiotemporal dynamics governed by PDEs

In this section, the HybridNDiff-UQ is utilized to model spatiotemporal dynamics with quantified uncertainty, which are also spatiotemporal fields. Specifically, its efficacy is assessed on a reaction-diffusion system governed by the following PDEs,

(17a) $${\dot{v}}_{1}=D_{1}\nabla^{2}v_{1}+s_{1}\left(v_{1},v_{2}\right),$$

(17c) $${\dot{v}}_{2}=D_{2}\nabla^{2}v_{2}+s_{2}\left(v_{1},v_{2}\right),$$

where $v_{1},v_{2}\in R^{n_{x}\times n_{y}}$ are the state variables over a discretized 2-D domain, characterized by $n_{x}\times n_{y}$ grid points in the x, y directions. The diffusion coefficients are set as $D_{1}=2.8\times 10^{-4}$ and $D_{2}=5.0\times 10^{-2}$. The source terms $s_{1}(v_{1},v_{2})=v_{1}-v_{1}^{3}-v_{2}-0.005$ and $s_{2}(v_{1},v_{2})=10(v_{1}-v_{2})$ define the reaction dynamics. Neumann boundary conditions are applied in these simulations. Similar to ODE cases above, synthetic datasets for these PDEs are generated and perturbed with Gaussian random noises. We assume that the model forms for the reaction dynamics is partially known: $s_{1}$ is given, while $s_{2}(v_{1},v_{2})$ remains unknown. Therefore, in our HybridNDiff-UQ framework, the known $s_{1}(v_{1},v_{2})$ is encapsulated within an UT function, resulting in a known random function $𝒦(V)=UT(s_{1})(V):R^{4}\Rightarrow R^{2}$, which is seamlessly integrated with Bayesian neural networks ${𝒱}_{bnn}(V):R^{4}\Rightarrow R^{2}$ to learn the missing reaction physics. The diffusion operator is encoded as known convolution operations using finite difference method (FDM),

(18) $$\nabla^{2}v(i,j)=\frac{v(i+1,j)+v(i-1,j)-2v(i,j)}{\Delta x^{2}}\;\;+\frac{v(i,j+1)+v(i,j-1)-2v(i,j)}{\Delta y^{2}},$$

where v(i+1,j),v(i−1,j), v(i+1,j), and v(i−1,j) are neighbouring values of v(i,j) post-discretization. Now, the discretized diffusion term as a linear combination of adjacent spatial points leads to the output mean and variance as

(19a) $$2\mu_{\nabla^{2}V}(i,j)=\frac{V(i+1,j)+V(i-1,j)-2V(i,j)}{\Delta x^{2}}\;\;+\frac{V(i,j+1)+V(i,j-1)-2V(i,j)}{\Delta y^{2}},$$

(19b) $$\Sigma_{\nabla^{2}V}^{2}(i,j)=\frac{\Sigma_{V(i+1,j)}^{2}+\Sigma_{V(i-1,j)}^{2}+4\Sigma_{V(i,j)}^{2}}{\Delta x^{4}}+\frac{\Sigma_{V(i,j+1)}^{2}+\Sigma_{V(i,j-1)}^{2}+4\Sigma_{V(i,j)}^{2}}{\Delta y^{4}}.$$

Furthermore, diffusion coefficients $D_{i}$ are considered as unknown variables and kept trainable throughout all test cases, unless specifically mentioned otherwise. The Hybrid models are typically employed in scenarios with limited, indirect data. To replicate such conditions, unless otherwise stated, we provide training data only for the $v_{2}$ variable and exclude any labels for $v_{1}$. Training is conducted on a specific trajectory within the timeframe $\left[0:\Delta t:T^{\prime }\right]$, thus $𝒟=(v_{2}\in R^{n_{x}\times n_{y}}\times [0:\Delta t:T^{\prime }]$, where $T^{\prime }<T$. The model’s predictive performance is then evaluated in the test region $\left(v_{1}\in R^{n_{x}\times n_{y}}\times [0:\Delta t:T],v_{2}\in R^{n_{x}\times n_{y}}\times (T^{\prime }:\Delta t:T]\right)$.

#### 3.2.1. Dynamical forecasting with spatiotemporal uncertainty estimation

**Fig. 9.**
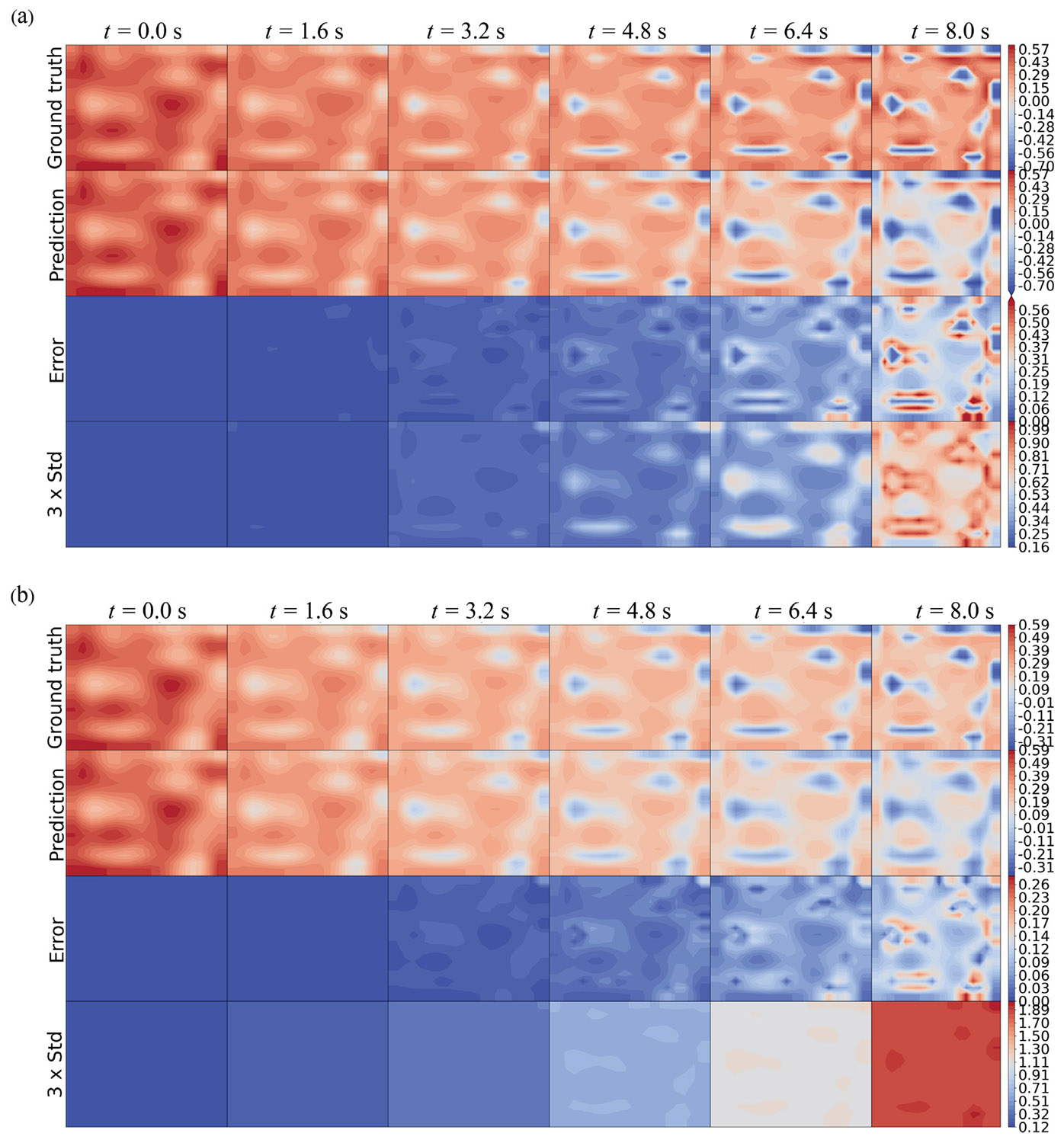
Comparison of the HybridNDiff-UQ model’s prediction (2nd row) with UQ (4th row) against the ground truth (1st row) for variable $v_{1}$ (a) and $v_{2}$ (b) over time. Training data: $𝒟=(v_{2}\in {\left(R\right)}^{20\times 20}\times [0:0.01:4\;s])$, with testing in $\left(v_{1}\in {\left(R\right)}^{20\times 20}\times [0:0.01:8\;s],v_{2}\in {\left(R\right)}^{20\times 20}\times (4:0.01:8\;s]\right)$.

**Fig. 10.**
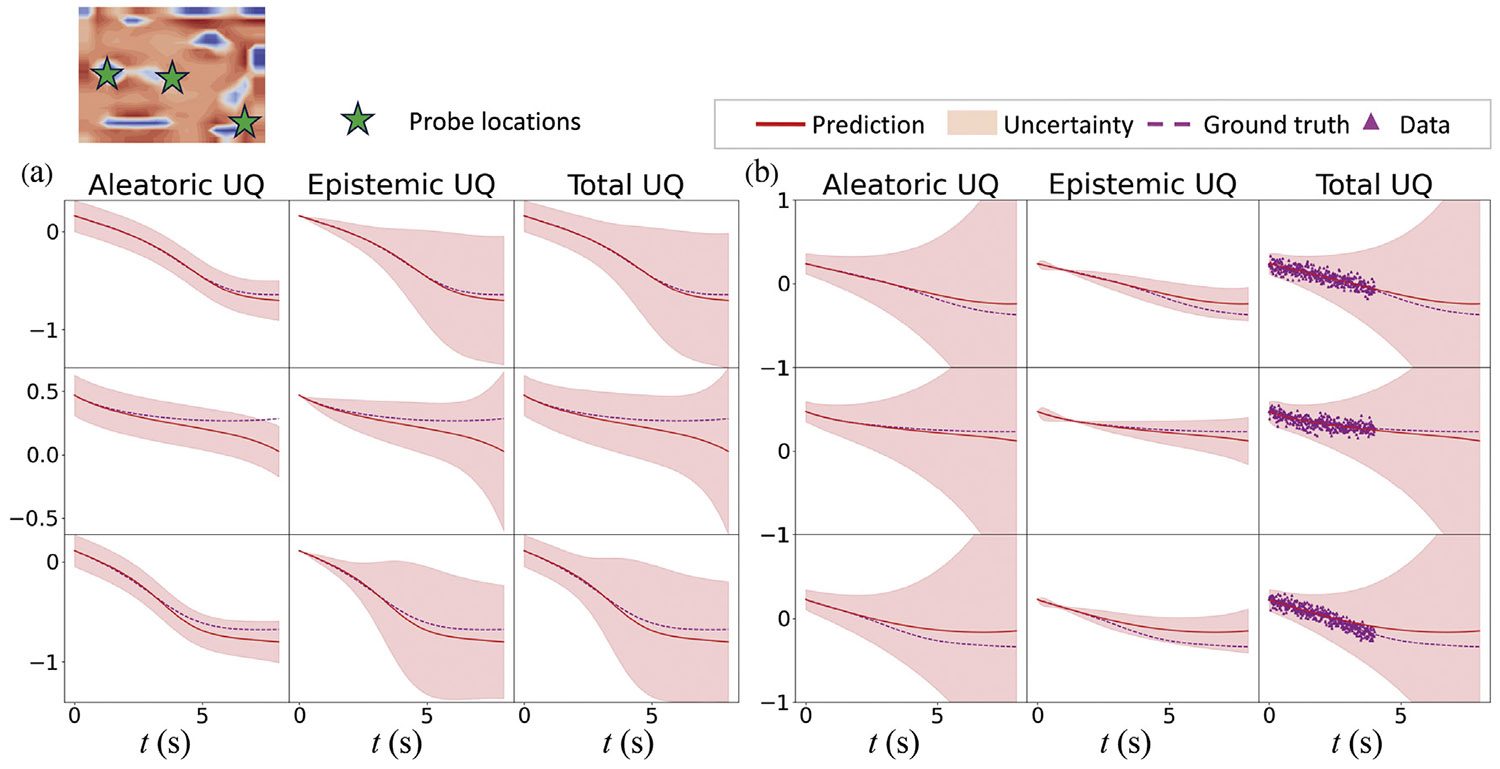
Comparison of the HybridNDiff-UQ model’s prediction with UQ against the ground truth for variable $v_{1}$ (a) and $v_{2}$ (b) at three spatial locations over time. Training data: $𝒟=(v_{2}\in {\left(R\right)}^{20\times 20}\times [0:0.01:4\;s])$, with testing in $\left(v_{1}\in {\left(R\right)}^{20\times 20}\times [0:0.01:8\;s],v_{2}\in {\left(R\right)}^{20\times 20}\times (4:0.01:8\;s]\right)$.

To investigate the spatiotemporal behavior of predicted uncertainty, we trained the HybridNDiff-UQ model using the data of a single trajectory for up to 4 s on a 20 × 20 grid, i.e., $𝒟=(v_{2}\in {\left(R\right)}^{20\times 20}\times [0:0.01:4\;s])$ and tested it in the forecast region (4 : 0.01 : 8 s]. For this analysis, the diffusion coefficients $D_{i}$ were assumed to be known. To clearly observe the spatial uncertainty prediction, the initial condition is defined by a Gaussian random field. The model was trained for 100 epochs, with results displayed in Figs. 9 and 10.

As we can see from Fig. 9, the prediction mean of both $v_{1}$ and $v_{2}$ agree with the ground truth reasonably well, while the prediction errors grow temporally. There is a strong correlation between the spatial prediction error (third row) and the estimated uncertainty (fourth row). Notably, no training data was provided for $v_{1}$, highlighting the model’s capability to learn the dynamics and estimate corresponding uncertainty in the absence of direct labels.

Fig.10 illustrates temporal uncertainty through a shaded region representing three times the standard deviation, signifying the confidence interval. This interval effectively captures the data uncertainty, which increases over time, consistent with the auto-regressive nature of the model where confidence diminishes with each subsequent time-step prediction. The aleatoric uncertainty for $v_{1}$ remains relatively constant throughout the forecast period since not direct labels are involved for $v_{1}$. In contrast, the data uncertainty for $v_{2}$ escalates over time, a consequence of the larger diffusion coefficient $D_{2}$. Since data for $v_{2}$ was provided during training, the epistemic uncertainty for $v_{2}$ is comparatively lower than that for $v_{1}$. These results affirm that the HybridNDiff-UQ model successfully captures the spatiotemporal behavior of predicted uncertainty.

#### 3.2.2. Inference and generalization capability of HybridNDiff-UQ model

**Fig. 11.**
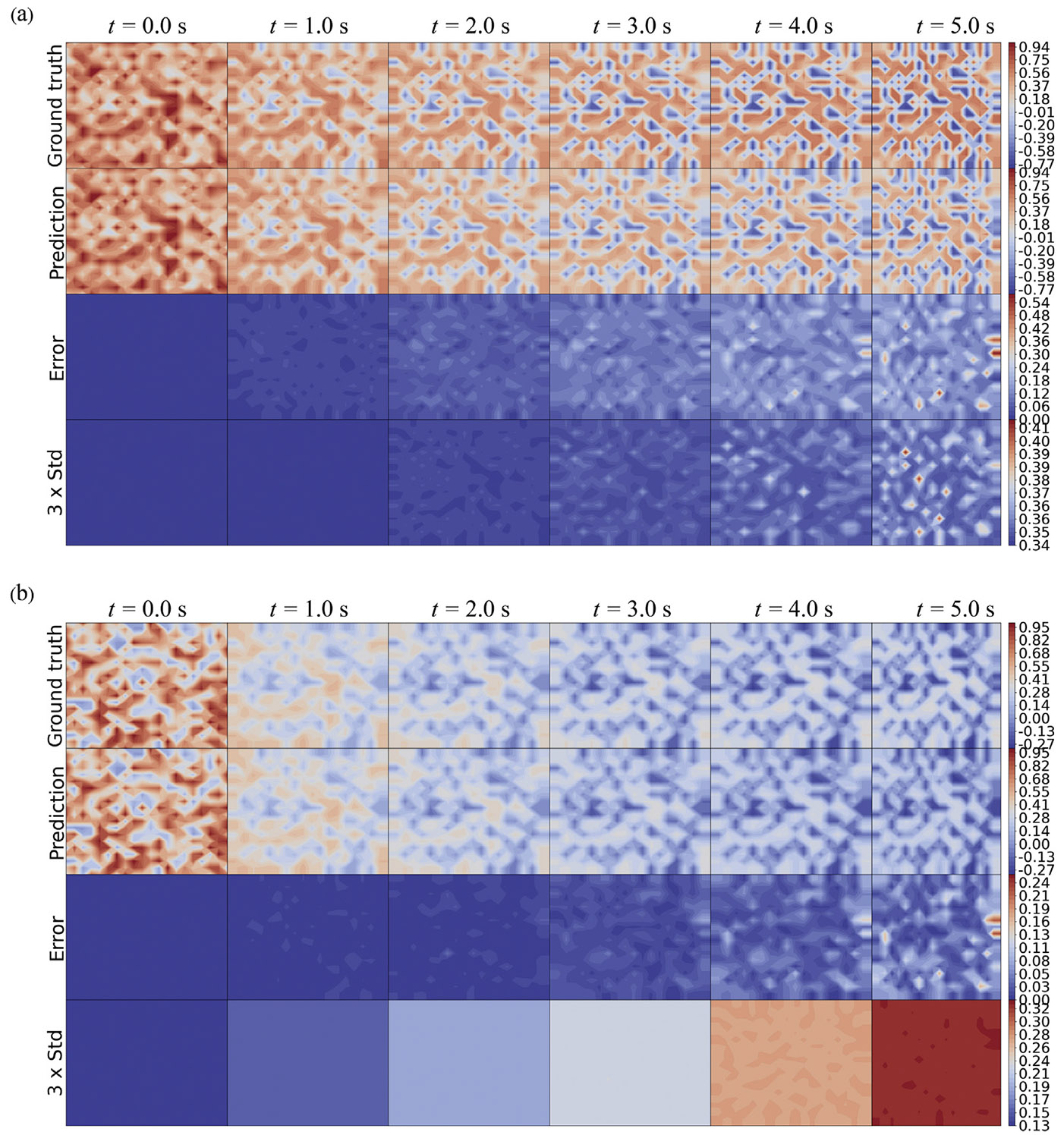
Comparison of the HybridNDiff-UQ model’s prediction (2nd row) with UQ (4th row) against the ground truth (1st row) for $v_{1}$ (a) and $v_{2}$ (b) over time. Training data: $𝒟=(v_{2}\in {\left(R\right)}^{20\times 20}\times [0:0.01:3.5\;s])$, with testing in $\left(v_{1}\in {\left(R\right)}^{20\times 20}\times [0:0.01:3.5\;s],v_{2}\in {\left(R\right)}^{20\times 20}\times (3.5:0.01:8\;s]\right)$.

**Fig. 12.**
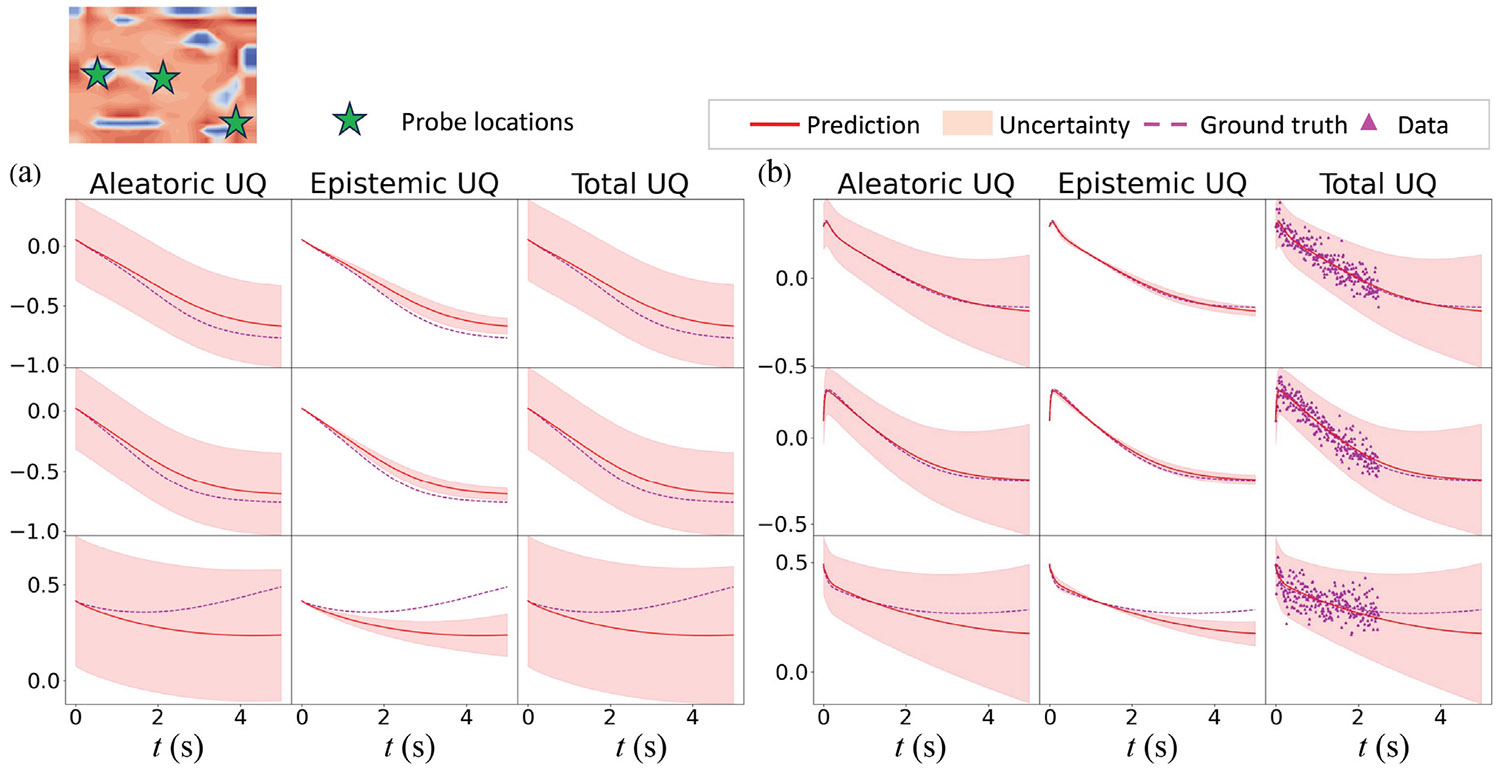
Comparison of the HybridNDiff-UQ model’s prediction with UQ against the ground truth for $v_{1}$ (a) and $v_{2}$ (b) at three spatial locations over time. Training data: $𝒟=(v_{2}\in {\left(R\right)}^{20\times 20}\times [0:0.01:3.5\;s])$, with testing in $\left(v_{1}\in {\left(R\right)}^{20\times 20}\times [0:0.01:3.5\;s],v_{2}\in {\left(R\right)}^{20\times 20}\times (3.5:0.01:5\;s]\right)$.

The inference capability of the HybridNDiff-UQ model is evaluated through an experimental study where the diffusion coefficients $D_{i}$ are assumed to be unknown and trainable. The model is trained on a single trajectory for up to 3.5 s $𝒟=(v_{2}\in {\left(R\right)}^{20\times 20}\times [0:0.01:3.5\;s])$ and then tested in the forecast region (3.5 : 0.01 : 5 s]. After 300 epochs of training, the outcomes are presented in Figs. 11 and 12.

The results indicate strong prediction performance by the model, as the prediction mean agree with the ground truth well for both variables. As depicted in Fig. 11, the predicted spatial uncertainty aligns well with expectations, which correlate with the prediction errors. The confidence interval shown in Fig. 12 effectively encapsulates the data scattering. Moreover, in line with prior observations, there is a notable increase in model-form uncertainty over time, further validating the model’s effectiveness in dynamic modeling with UQ.

**Fig. 13.**
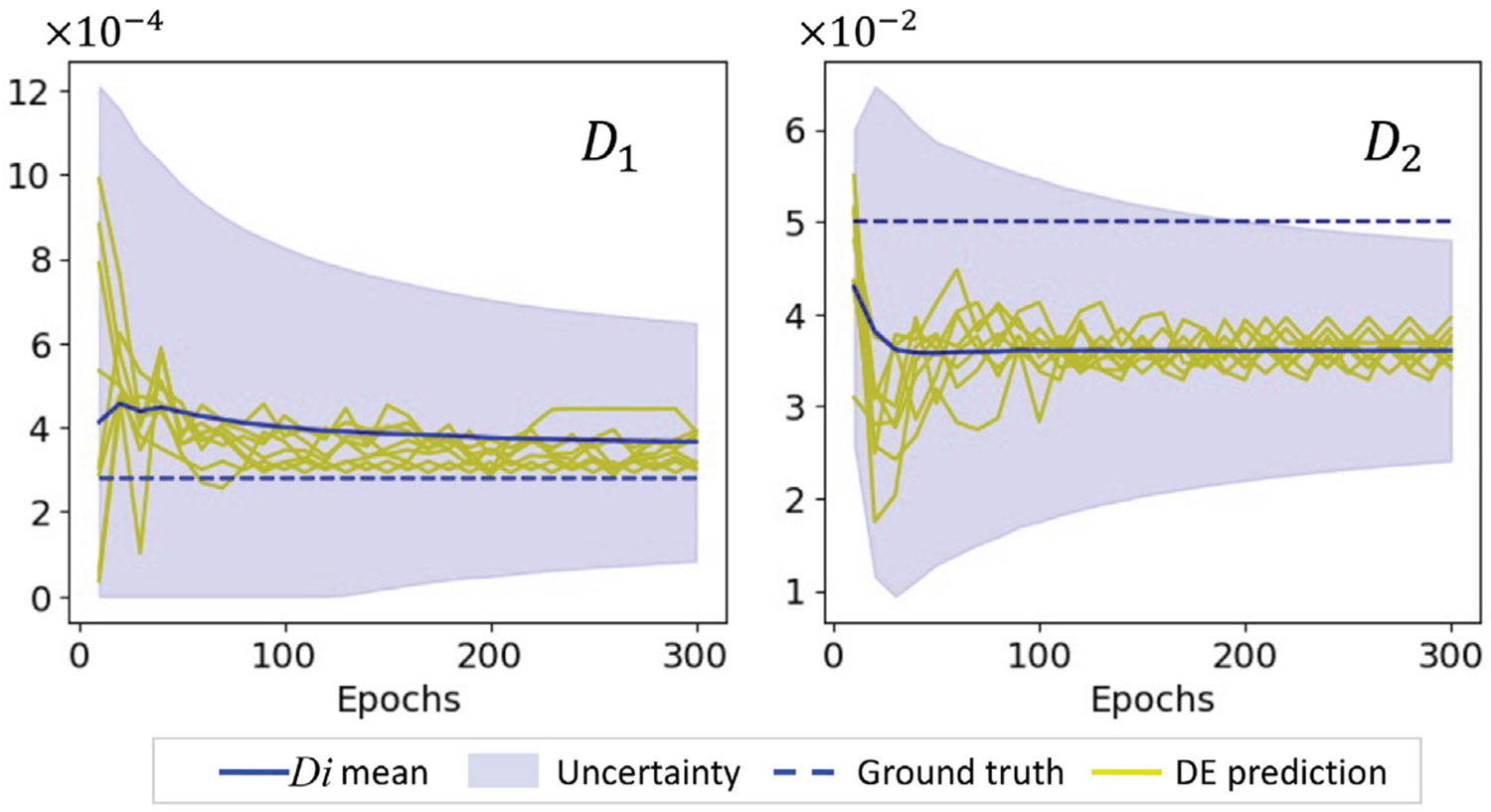
HybridNDiff-UQ inferred diffusion coefficients ($D_{1}$ and $D_{2}$) with quantified uncertainty over training epochs. DE represents ensembles of inferred diffusion coefficients.

In this case, the model accurately predicts the dynamics of the unobserved state $v_{1}$ and also successfully infers the unknown diffusion coefficients $D_{1}$ and $D_{2}$ during the training process, as shown in Fig. 13. Hybrid models are often susceptible to aliasing errors, particularly when trained with limited data. This issue can lead to inaccuracies in both the learned surrogate functions and the inferred coefficients, with the magnitude of errors typically increasing as the size of training data diminishes. In response to this challenge, the HybridNDiff-UQ model is designed to estimate errors in inferred parameters, thereby providing a more robust analysis. Fig. 13 exemplifies the model’s capability in quantifying uncertainty associated with the diffusion coefficients throughout the training process. This uncertainty, indicative of the aliasing effect, is observed to decrease as training advances. While it is not possible to guarantee absolute accuracy in the inferred diffusion coefficients due to the inverse and ill-posed nature of the problem, the HybridNDiff-UQ model enables a reasonably confident estimation. The fidelity of these inferences is contingent upon the dominant physics captured in the data, along with other influential factors, which will be elaborated upon in the Discussion section.

**Fig. 14.**
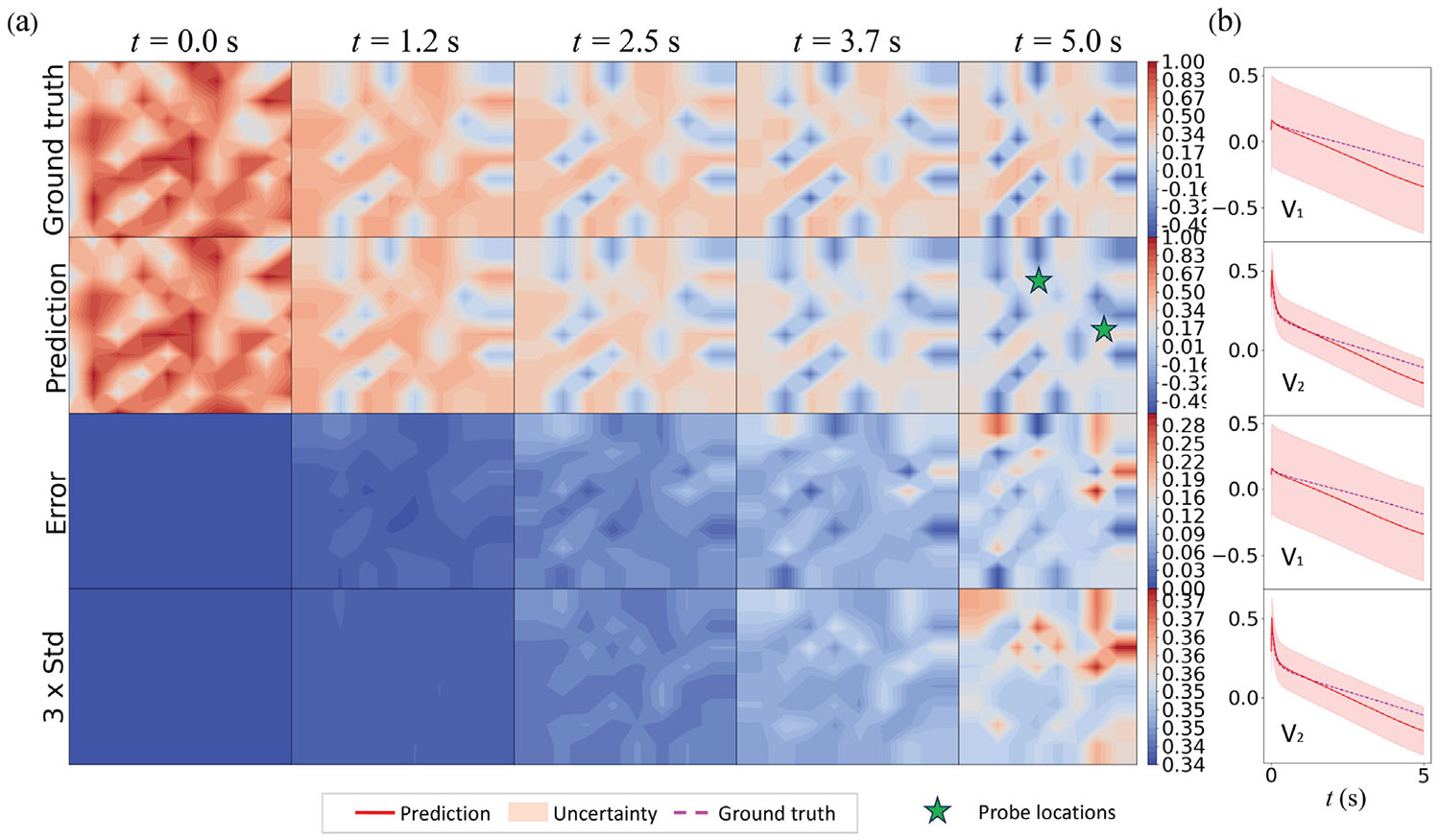
HybridNDiff-UQ testing results on an unseen randomly generated initial field, on a 10 × 10 grid with time-step of 0.02 s.

**Fig. 15.**
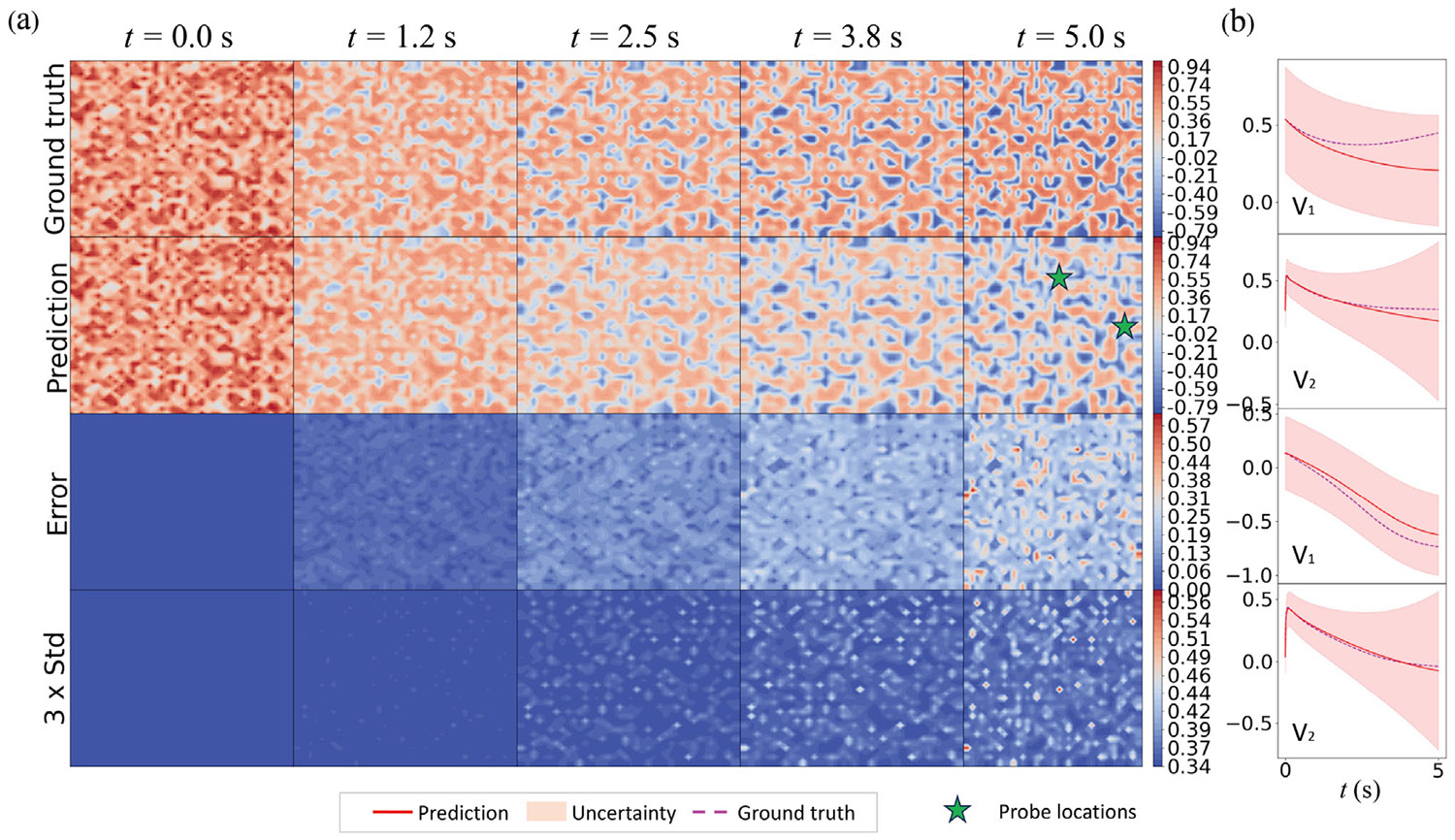
HybridNDiff-UQ testing results on an unseen randomly generated initial field, on a 40 × 40 grid with time-step of 0.001 s.

To evaluate the HybridNDiff-UQ model’s generalizability, we performed tests on various grid sizes and time steps, using a randomly generated initial conditions, which is distinct from the one used in the training phase. Fig. 14 illustrates the test results on a 10 × 10 grid with a time step of 0.02 s, while Fig. 15 presents the test results for a 40 × 40 grid with a time step of 0.001 s. Across these tests, the model’s predictions agree with the references reasonably well, demonstrating great generalizability. Notably, regions with higher deviation from the ground truth correspond to increased uncertainty in the model’s predictions. This observation underscores the model’s capability of quantifying uncertainty, even under varying testing conditions. It is crucial to highlight that, despite potential inaccuracies in the inferred diffusion coefficients, the trained surrogate model consistently demonstrates its ability to provide reliable predictions with quantified uncertainty.

#### 3.2.3. Correcting discretization errors and speedup

In hybrid neural solvers, employing coarse spatio-temporal resolution is a common practice to enhance inference efficiency. However, this approach can introduce significant discretization errors in the PDE-integrated components, potentially affecting the accuracy of solutions. Within our HybridNDiff-UQ framework, this issue can be addressed by trainable neural network blocks, which are designed not only to learn and model unknown physical phenomena but also to concurrently correct for the discretization errors inherent in the coarser resolution. This dual capability allows the HybridNDiff-UQ model to maintain solution accuracy while benefiting from the computational efficiency of a coarser grid and larger timesteps. In this context, we explored the efficacy of the HybridNDiff-UQ model operating on coarse spatiotemporal resolutions. To this end, no structural modifications are needed, and the model’s ${𝒱}_{nn}$ component serves a dual role: it acts as a surrogate for unknown functions and corrects solutions on coarser grids. In our evaluation, the model was trained on a coarse 20 × 20 grid with a time step of 0.01 s, using data sampled from a high-resolution solution on a 200 × 200 grid with a finer time step of 0.0005 s. This approach allowed us to investigate the model’s ability to replicate fine-grid precision on a coarser grid. The training data covered a single trajectory up to 3.7 s, denoted as $𝒟=(v_{1},v_{2}\in {\left(R\right)}^{20\times 20}\times [0:0.01:3.5\;s]∼{\left(R\right)}^{200\times 200}\times [0:0.0005:3.5\;s])$, and the model’s predictions were subsequently assessed in the forecast period of 3.7 s to 5 s.

**Fig. 16.**
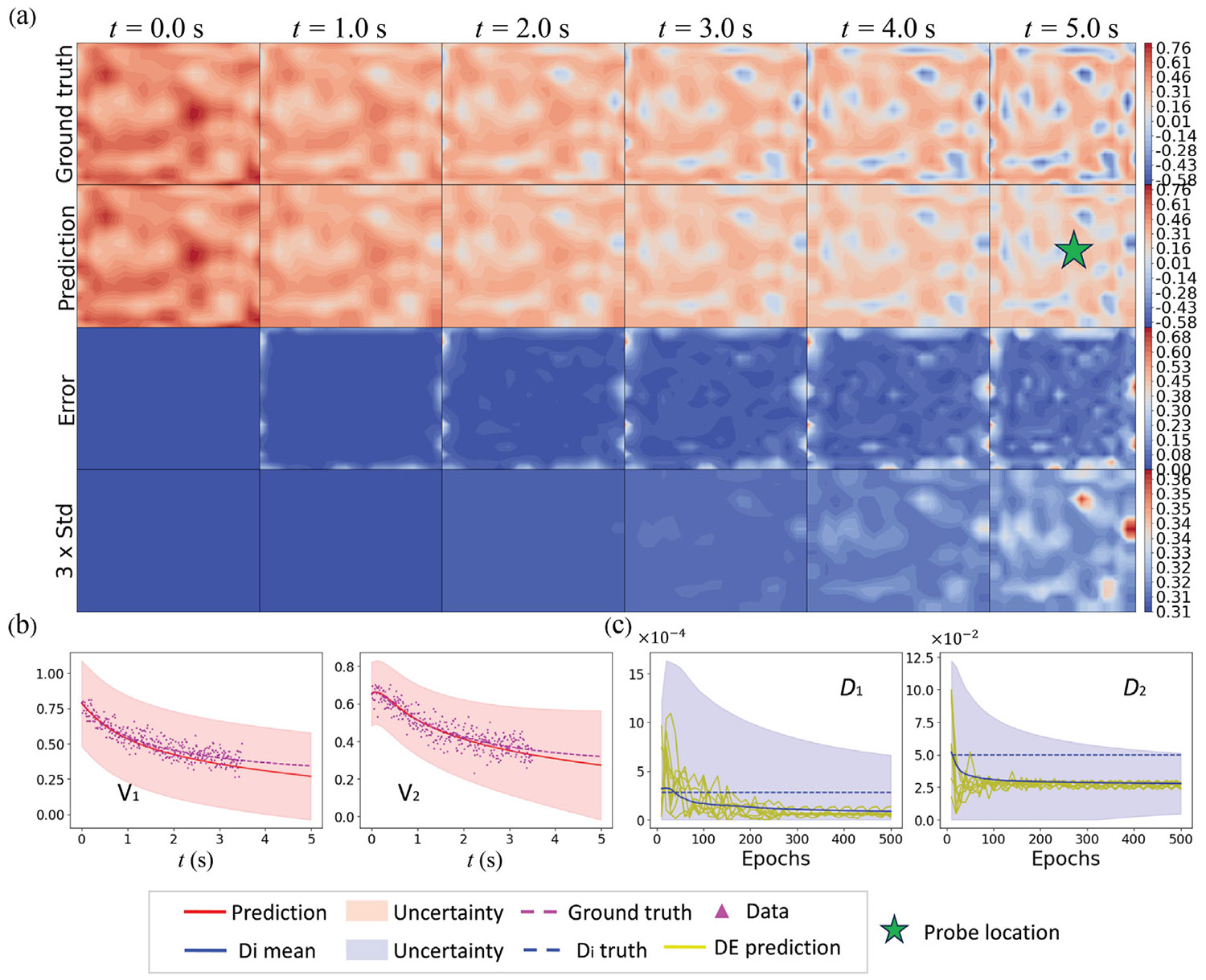
Compares the HybridNDiff-UQ model’s predictions and UQ on a 20 × 20 grid with a time step of 0.01 s against the ground truth generated on a 200 × 200 grid with a time step of 0.0005 s. Training data spans $𝒟=(v_{1},v_{2}\in {\left(R\right)}^{20\times 20}\times [0:0.01:3.5\;s]∼{\left(R\right)}^{200\times 200}\times [0:0.0005:3.5\;s])$ with testing in the $\left(v_{1},v_{2}\in {\left(R\right)}^{20\times 20}\times [3.5:0.01:5\;s]∼{\left(R\right)}^{200\times 200}\times (3.5:0.0005:5\;s]\right)$ region.

Post-training for 500 epochs, the HybridNDiff-UQ model exhibited commendable performance within the training range. The results, presented in Fig. 16, showed reasonable prediction accuracy. However, a deviation from the ground truth was observed in the extrapolation region, where the model predicted a notably higher level of uncertainty, reflecting its recognition of increased error potential. This ability to predict with heightened uncertainty in less certain regions demonstrates the model’s robustness, offering fine-grid level accuracy on coarser grids and thus reducing computational load. The efficiency of the HybridNDiff-UQ model, in terms of inference time and memory usage for both fine and coarse simulations, is detailed in Table 2, highlighting its capability to produce high-fidelity results with lower computational demands and memory requirements.

### 3.3. Identifiability of unknown physical parameters

**Fig. 17.**
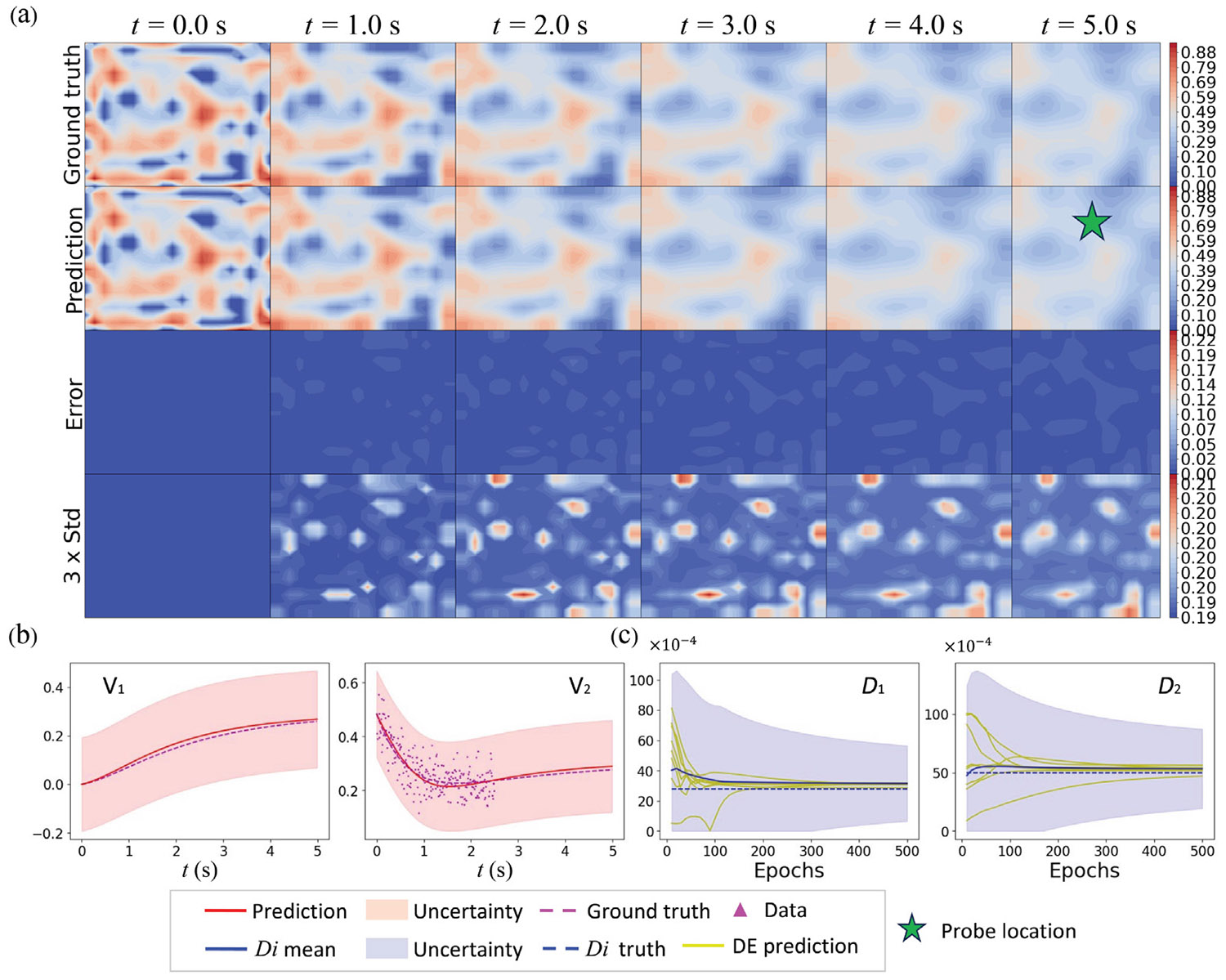
HybridNDiff-UQ test predictions with UQ, where diffusion coefficients are inferred simultaneously.

The identifiability of physical parameters (e.g., diffusion coefficients) within the HybridNDiff-UQ framework, presents intriguing insights into the model’s capability and limitations. As shown in cases presented above, a notable issue is the relatively low accuracy of inferred mean diffusion coefficients and associated high prediction uncertainty. This phenomenon can be attributed to the training data’s dynamics being primarily driven by reaction physics, reducing the influence of the diffusion term on the overall dynamics. Therefore, the diffusion coefficients are less sensitive and difficult to be inferred accurately. We hypothesized that when the dynamics are more substantially influenced by the diffusion process, the model’s ability to accurately infer diffusion coefficients would improve. To test this hypothesis, a simulation with enhanced diffusion prominence was conducted, setting $D_{1}=2.8\times 10^{-3}$ and $D_{2}=5.0\times 10^{-3}$. This modification aimed to shift the focus towards the diffusion process. After 300 epochs of training, the results, as depicted in Fig. 17, indicated a notable improvement in parameter inversion accuracy. The model’s estimated confidence intervals closely captured the data/aleatoric uncertainty, and the inferred diffusion coefficients were more aligned with the ground truth. Furthermore, a decrease in model uncertainty was observed with each training epoch. These observations substantiate our hypothesis, emphasizing that the model’s inferential accuracy is heavily dependent on the representation of governing physical processes in the training data. In scenarios where diffusion processes dominate, the model has enhanced capability in accurately inferring diffusion coefficients.

## 4. Conclusion

In this study, we have introduced HybridNDiff-UQ, a novel approximate Bayesian learning framework for quantifying uncertainty in physics-integrated hybrid neural differentiable models. This methodology, blending ensemble Bayesian learning with nonlinear transformations, has demonstrated substantial potential in effectively capturing and quantifying uncertainties associated with data-driven modeling of complex physical systems. Through rigorous evaluation on both ordinary and partial differential equations, the HybridNDiff-UQ model has demonstrated its proficiency in diverse aspects of UQ. Notably, it has accurately provided lower confidence levels for predictions in regions with sparse training data, effectively capturing the growth of uncertainty over time due to error accumulation. In scenarios involving parametric extrapolation, the model adeptly indicated increased uncertainty, particularly when initial conditions diverged from those in the training set. For spatiotemporal problems governed by PDEs, the HybridNDiff-UQ model can be trained with partial observational data, delivering reasonable predictions of spatiotemporal uncertainty. Its capacity to simultaneously infer physical parameters with quantified uncertainty, especially in the presence of aliasing errors, was particularly noteworthy. Furthermore, we also showcased the HybridNDiff-UQ model’s ability to provide accurate predictions on coarse grids with reduced computational demands, underscoring its potential in practical applications where computational efficiency is critical. In sum, this work marks a crucial stride forward in the field of UQ for hybrid neural differentiable models that integrate physics-based numerical components with deep learning models using differentiable programming. This advancement holds great promise for refining physics-informed, data-driven decision-making processes across a wide array of scientific and engineering disciplines, fostering more accurate, robust, and trustworthy models for complex systems analysis.

Overall, this research represents a significant advancement in the field of uncertainty quantification for physics-integrated hybrid deep neural differentiable models. The successful integration of ensemble learning and nonlinear transformation provides a robust framework for enhancing prediction reliability and interpretability. The proposed method holds immense potential for improving hybrid simulation-based decision-making processes across various scientific and engineering domain. One particularly promising direction is its application to complex fluid modeling in CFD. For example, HybridNDiff-UQ could be used to learn physics-informed surrogate operators for unclosed terms in Reynolds-averaged Navier Stokes (RANS) equations, such as the Reynolds stress tensor. Embedding such learned closures within a differentiable solver would enable end-to-end training while preserving numerical consistency and physical fidelity. The ability to quantify both aleatoric and epistemic uncertainty further supports risk-aware modeling in turbulence-dominated flows. While challenges remain, such as data requirements from high-fidelity simulations and generalization across flow regimes, the scalability and uncertainty-awareness of HybridNDiff-UQ make it a compelling foundation for advancing data-driven turbulence modeling.

## Figures and Tables

**Table 1:** Test cases to examine the model’s efficacy in accurately estimating uncertainties under varying data availability and measurement error conditions.

Case	Data 𝒟	Description
1	$\left(x_{1},x_{2}\in {\left(R\right)}^{1}\times [0:0.1:20\;s]\right)$	Complete data for both variables are provided
2	$\left(x_{1},x_{2}\in {\left(R\right)}^{1}\times [0:1:20\;s])(x_{1},x_{2}\in {\left(R\right)}^{1}\times [0:0.1:10\;s])(x_{1},x_{2}\in {\left(R\right)}^{1}\times [5:0.1:20\;s]\right)$	Partial data for both variables are provided
3	$\left(x_{1}\in {\left(R\right)}^{1}\times [0:0.1:20\;s])(x_{2}\in {\left(R\right)}^{1}\times [0:1:20\;s]\right)$	Partial data for only one variable is provided
4	$\left(x_{1},x_{2}\in {\left(R\right)}^{1}\times [0:0.1:20\;s])(\mathrm{var}=0.1^{2}\;\text{and}\;\mathrm{var}=1.0^{2}\right)$	Different measurement error are provided

**Table 2:** Inference time and memory consumption.

Inference	CPU time	GPU time	Memory
${\left(R\right)}^{2\times 20\times 20}\times [0:0.01:5\;s]$	0.67 s	0.66 s	12 MB
${\left(R\right)}^{2\times 200\times 200}\times [0:0.0005:5\;s]$	8.8 s	1.15 s	33 GB

**Fig. C1.**
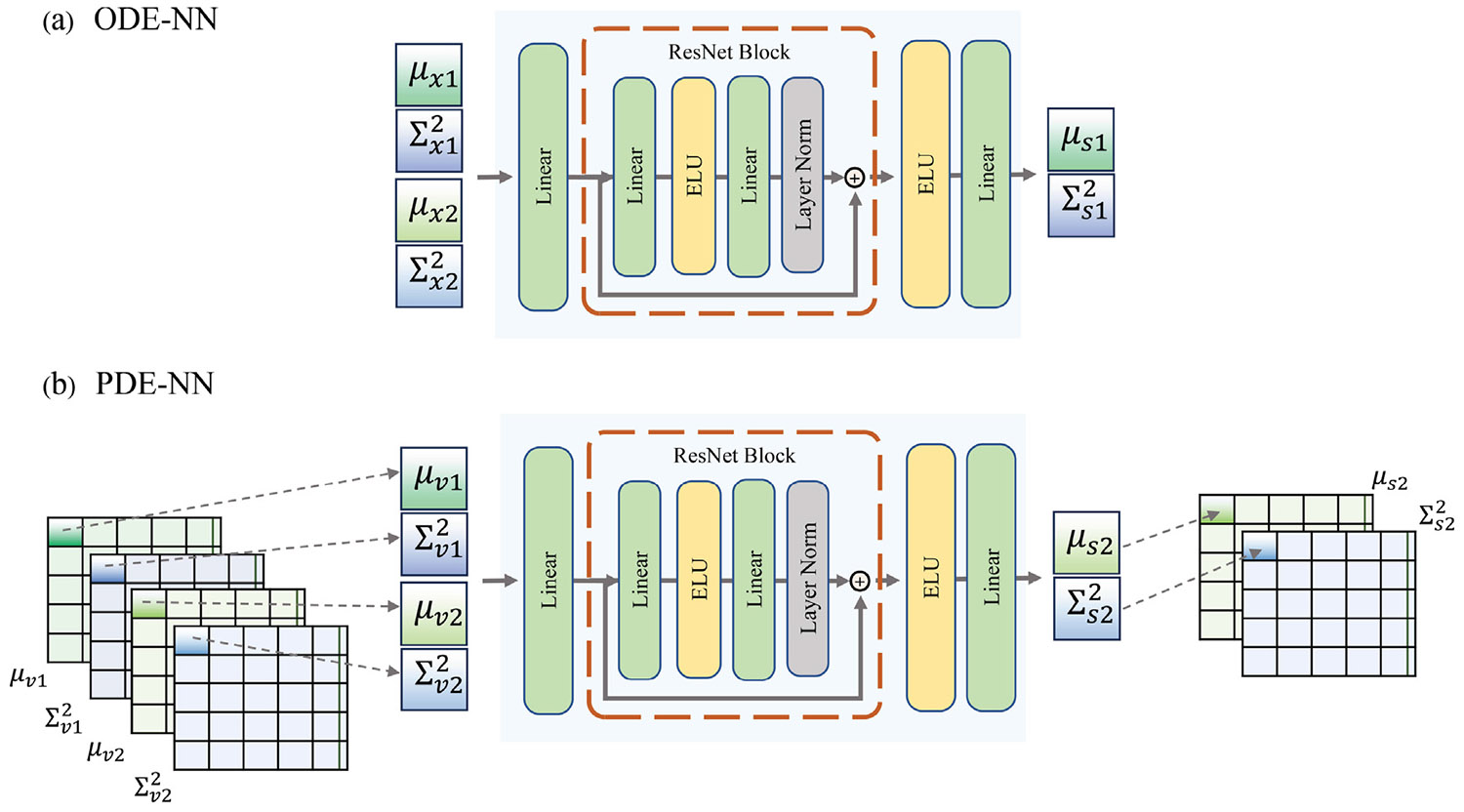
Neural network architecture used in HybridNDiff-UQ model.

**Fig. E1.**
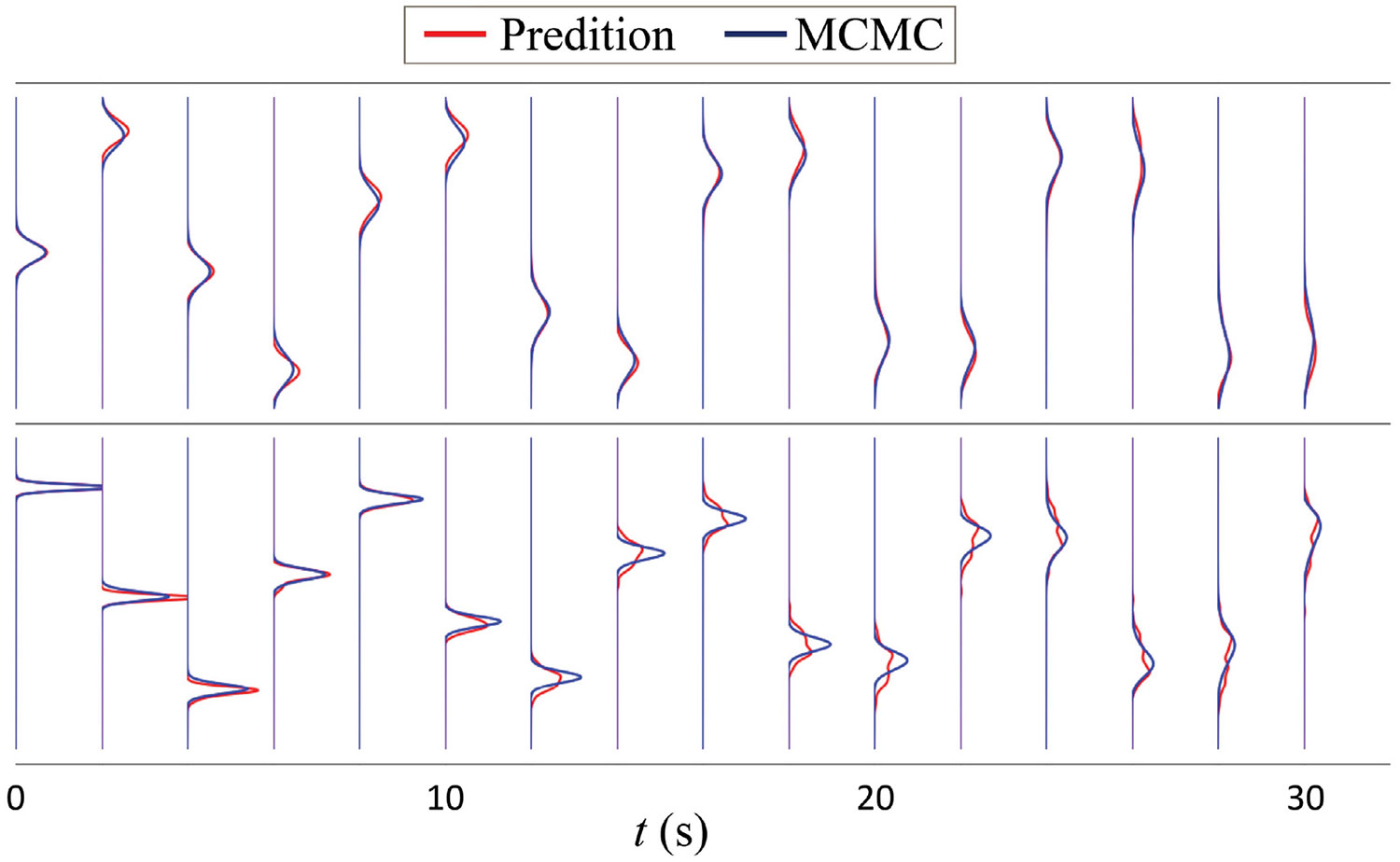
Compares the HybridNDiff-UQ model’s distribution prediction against HMC distribution. Training data spans $𝒟=(x_{1},x_{2}\in {\left(R\right)}^{1}\times [0:0.1:20\;s])$ with testing in the $\left(x_{1},x_{2}\in {\left(R\right)}^{1}\times [20:0.1:30\;s]\right)$ region.

**Fig. E2.**
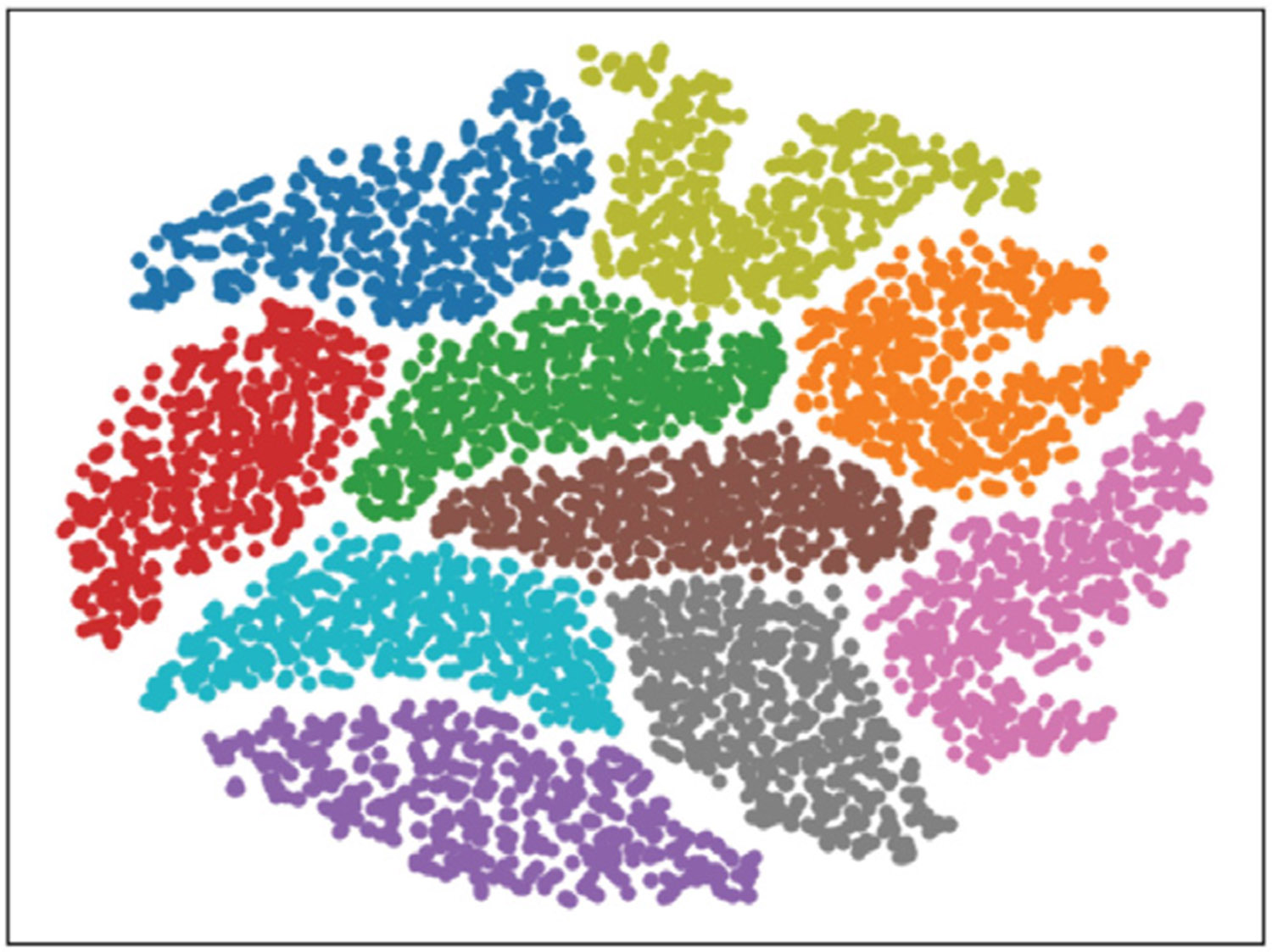
Tranable parameters projection using t-SNE method.

**Fig. E3.**
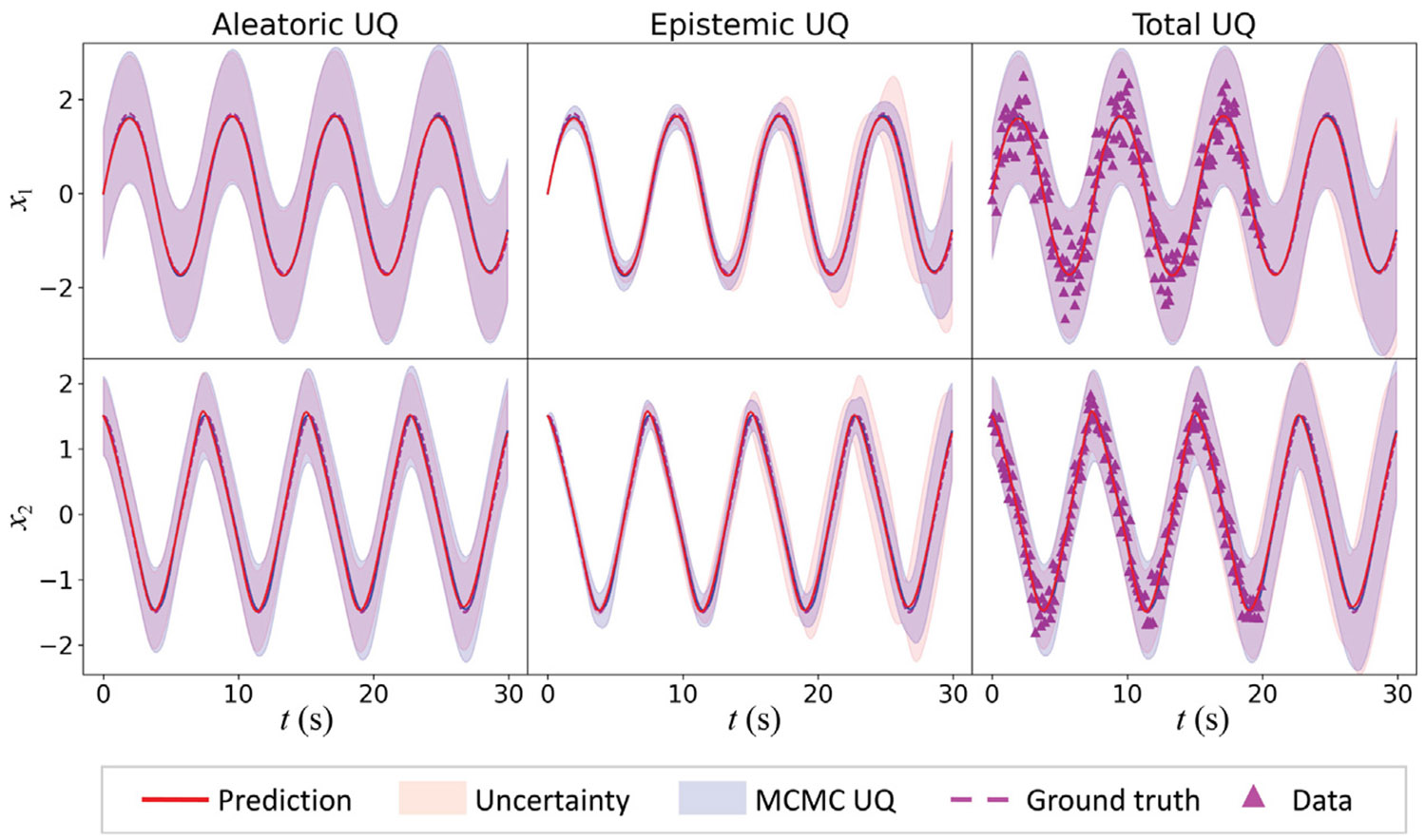
Compares the HybridNDiff-UQ model’s prediction and UQ against HMC method. Training data spans $𝒟=(x_{1},x_{2}\in {\left(R\right)}^{1}\times [0:0.1:20\;s])$ with testing in the $\left(x_{1},x_{2}\in {\left(R\right)}^{1}\times [20:0.1:30\;s]\right)$ region.

**Fig. E4.**
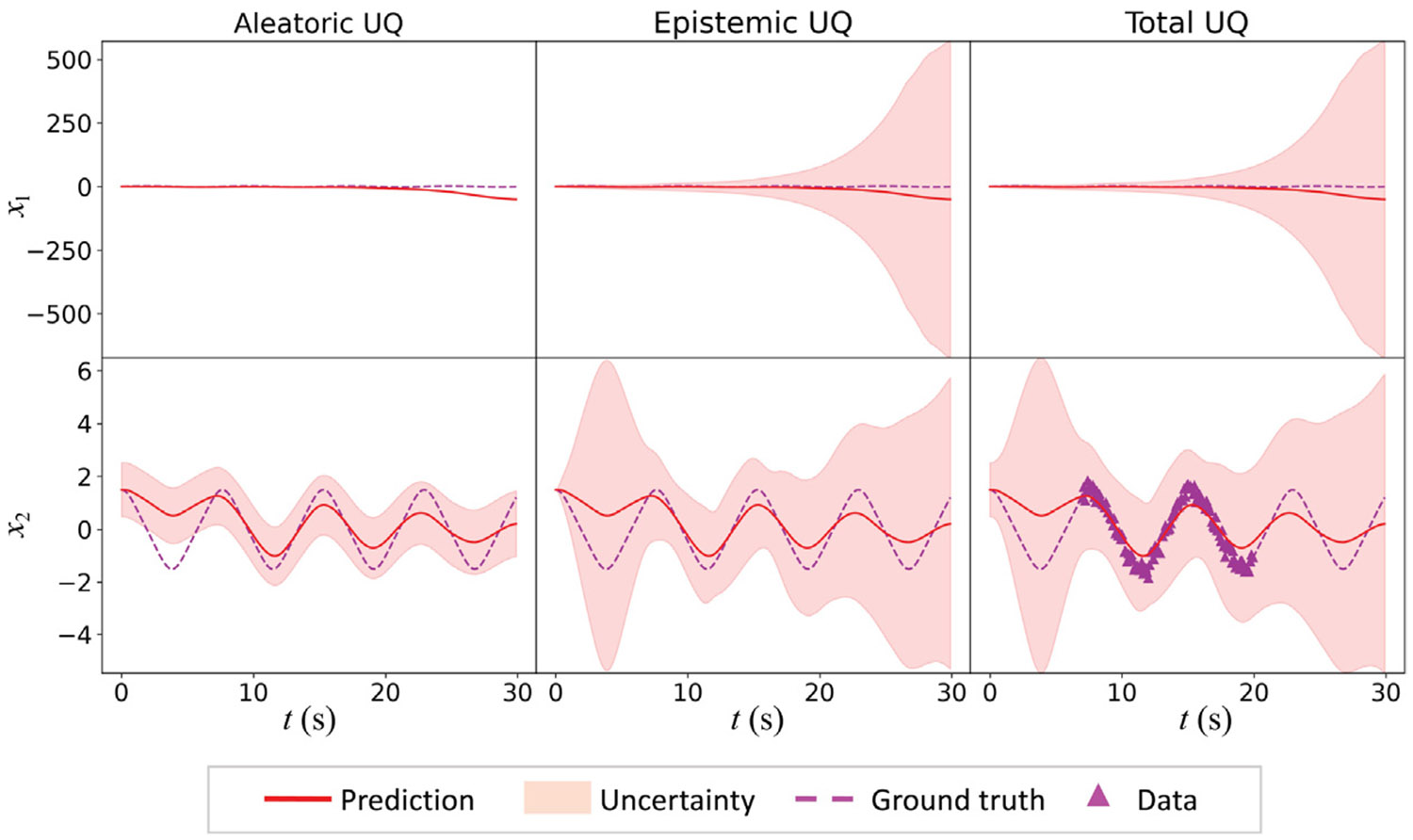
Comparison of the HybridNDiff-UQ model’s prediction with UQ against the ground truth. Training data is $𝒟=(x_{2}\in {\left(R\right)}^{1}\times [5:0.1:20\;s])$, with testing in $\left(x_{1}\in {\left(R\right)}^{1}\times [0:0.1:30\;s],x_{2}\in {\left(R\right)}^{1}\times [0:0.1:5\;s\cup (20:0.1:30\;s)\right]$ region.

**Fig. E5.**
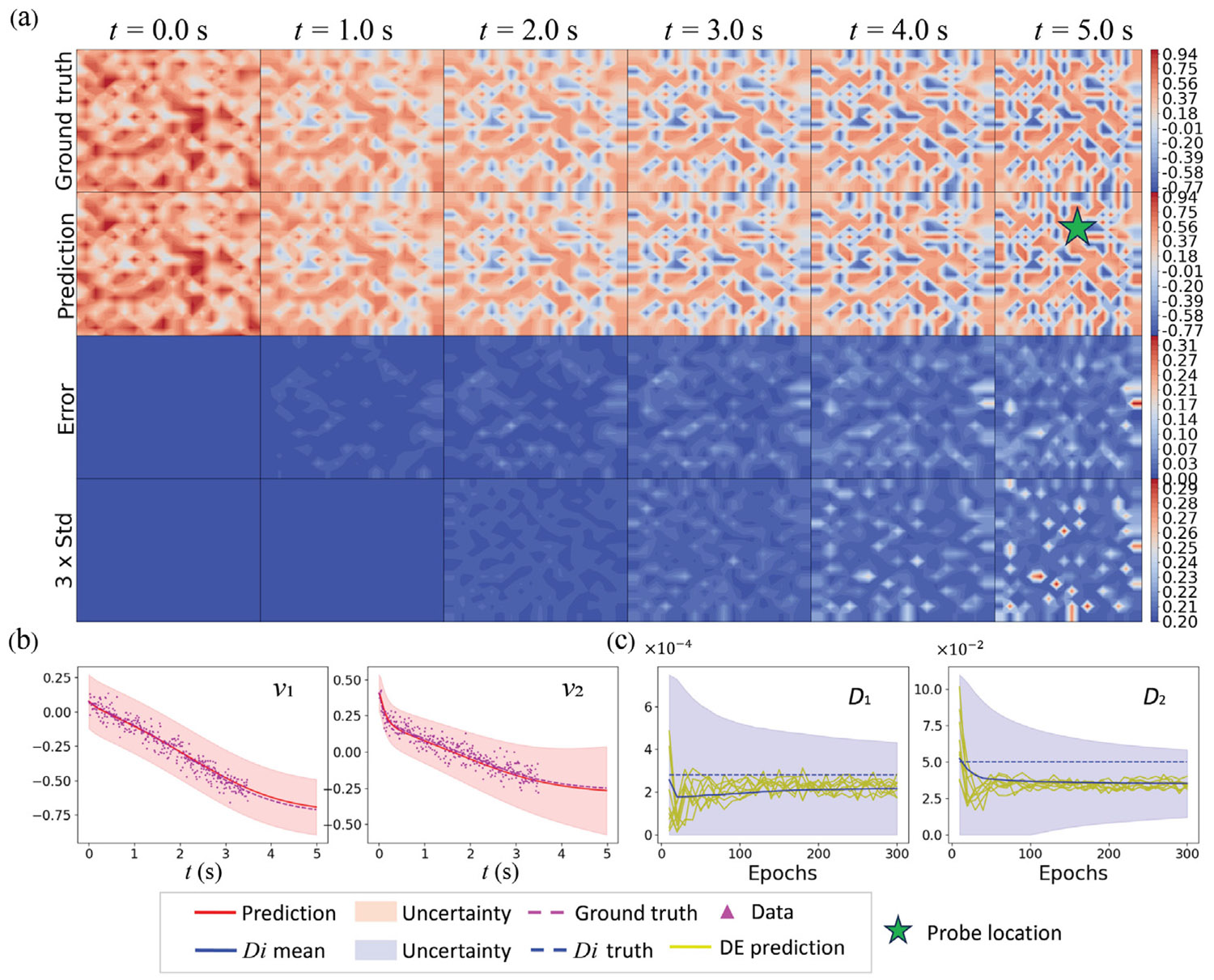
Compares the HybridNDiff-UQ model’s predictions and UQ on a 20 × 20 grid with a time step of 0.01 s against the ground truth. Training data spans $𝒟=(v_{1},v_{2}\in {\left(R\right)}^{20\times 20}\times [0:0.01:3.5\;s])$ with testing in the $\left(v_{1},v_{2}\in {\left(R\right)}^{20\times 20}\times (3.5:0.01:5\;s]\right)$ region.

**Fig. E6.**
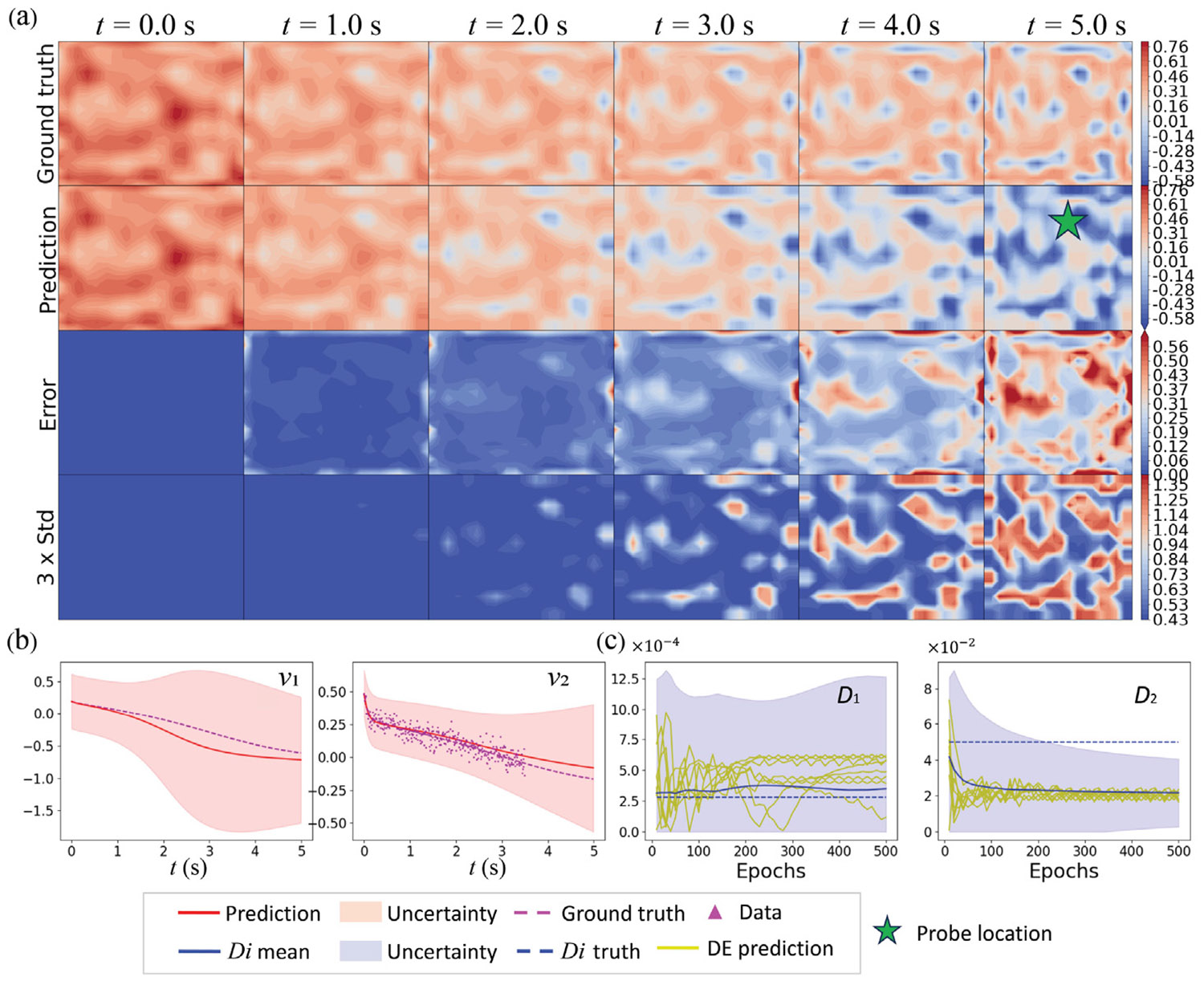
Compares the HybridNDiff-UQ model’s predictions and UQ on a 20 × 20 grid with a time step of 0.01 s against the ground truth generated on a 200 × 200 grid with a time step of 0.0005 s. Training data spans $𝒟=(v_{2}\in {\left(R\right)}^{20\times 20}\times [0:0.01:3.5\;s]∼{\left(R\right)}^{200\times 200}\times [0:0.0005:3.5\;s])$ with testing in the $\left(v_{1}\in {\left(R\right)}^{20\times 20}\times (0:0.01:5\;s])∼{\left(R\right)}^{200\times 200}\times [0:0.0005:5\;s],v_{2}\in {\left(R\right)}^{20\times 20}\times (3.5:0.01:5\;s])∼{\left(R\right)}^{200\times 200}\times (3.5:0.0005:5\;s]\right)$ region.

## Data Availability

All data needed to evaluate the conclusions in the paper are either present in the paper or can be regenerated by the code provided. The code required to reproduce these findings is available to download from https://github.com/jx-wang-s-group/HybridNDiff-UQ upon publication.

## Appendix A. Stochastic weight averaging Gaussian (SWAG)

The detailed SWAG [55] algorithm is described in this section. For a given SGD training trajectory $\theta_{\mathrm{traj}}={\left\{\theta_{i}\right\}}_{i=1}^{T}=\{\theta_{1},\theta_{2},\ldots ,\theta_{T}\}$, the SWAG algorithm formulates an approximate Gaussian distribution $𝒩(\theta_{\mathrm{SWA}},\frac{1}{2}(\Sigma_{\mathrm{diag}}+\Sigma_{\text{low-rank}}))$ for posterior, where the mean $\theta_{\mathrm{SWA}}$ is calculated as

(A1)
$$\theta_{SWA}=\overset{‾}{\theta }=\frac{1}{T}\sum_{i=1}^{T}\;\theta_{i},$$

and the diagonal covariance approximation $\Sigma_{\mathrm{diag}}$ of the posterior distribution is calculated as

(A2a)
$$\overset{-}{\theta^{2}}=\frac{1}{T}\sum_{i=1}^{T}\;\theta_{i}^{2},$$

(A2b)
$$\Sigma_{diag}=diag\left(\overset{-}{\theta^{2}}-\theta_{SWA}^{2}\right).$$

The sample covariance matrix of rank T for a given SGD training trajectory $\theta_{\mathrm{traj}}$ can be calculated using Eq. (A3a). However, storing the whole trajectory $\theta_{\mathrm{traj}}$ for a model with a large number of parameters will be an infeasible task. Therefore, the SWAG method approximates the sample covariance with a low rank r matrix as given in Eq. (A3b) and store deviation matrix $\hat{D}$ with columns $d_{i}=(\theta_{i}-{\bar{\theta }}_{i})$.

(A3a)
$$\Sigma =\frac{1}{T-1}\sum_{i=1}^{T}\;\left(\theta_{i}-\theta_{SWA}\right){\left(\theta_{i}-\theta_{SWA}\right)}^{T},$$

(A3b)
$$\Sigma_{low-rank\;}\approx \frac{1}{r-1}\sum_{i=T-r+1}^{T}\;\left(\theta_{i}-{\overset{‾}{\theta }}_{i}\right){\left(\theta_{i}-{\overset{‾}{\theta }}_{i}\right)}^{T}=\frac{1}{r-1}\sum_{i=T-r+1}^{T}\;\overset{ˆ}{D}{\overset{ˆ}{D}}^{T}.$$

Namely, instead of storing the whole trajectory, the posterior distribution statistics are updated while training according to the following algo [55]

(A4a)
$$\overset{-}{\theta }\leftarrow \frac{n\overset{-}{\theta }+\theta_{i}}{n+1},$$

(A4b)
$$\overset{-}{\theta^{2}}\leftarrow \frac{n\overset{-}{\theta^{2}}+\theta_{i}^{2}}{n+1},$$

(A4c)
$$\overset{ˆ}{D}\leftarrow concatenate\left(\overset{ˆ}{D}[:,2:],\theta_{i}-\overset{-}{\theta }\right),$$

and the following equation is utilized to obtain samples from $𝒩(\theta_{\mathrm{SWA}},\frac{1}{2}(\Sigma_{\mathrm{diag}}+\Sigma_{\text{low-rank}}))$ for prediction,

(A5)
$$\tilde{\theta }=\theta_{SWA}+\frac{1}{\sqrt{2}}\cdot \Sigma_{diag}^{1/2}z_{1}+\frac{1}{\sqrt{2(r-1)}}\overset{ˆ}{D}z_{2},z_{1}∼𝒩\left(0,I_{d}\right),z_{2}∼𝒩\left(0,I_{r}\right).$$

## Appendix B. Discretization of governing PDE for the stochastic state $V(\bar{x})$

Considering the probability distribution of all the random variables are diabolized Gaussian distribution, the mean and variance of the random variable, which is a linear combination of other random variables $Y=\sum_{i}c_{i}X_{i}$ is calculated as

(B1a)
$$\mu_{Y}=\sum_{i}\;c_{i}\mu_{Xi},$$

(B1b)
$$\Sigma_{Y}^{2}=\sum_{i}\;c_{i}^{2}\Sigma_{Xi}^{2}.$$

### B1. Discretization in time

Lets rewrite Eq. (13a)

$$\frac{\partial V_{\theta }(\overset{-}{x})}{\partial t}={ℱ}_{nn}\left[𝒦\left(V_{\theta }(\overset{-}{x});\lambda_{𝒦},\lambda_{𝒱}\right),{𝒱}_{nn}\left(V_{\theta }(\overset{-}{x});\lambda_{𝒦},\lambda_{𝒱},\theta_{nn}\right);\theta_{nn}\right],$$

as

(B2)
$$\frac{\partial }{\partial t}(V(x,t)|\theta )={ℱ}_{nn}(t,V(x,t)|\theta ).$$

Now to discretize Eq. (B2) in time, we approximate the spatiotemporal random state variable V(x,t) as a spatial random state variable at various times $\left\{0,\Delta t,2\Delta t,\ldots ,t,\ldots ,T_{r}\right\}$,

$$V(x,t)\approx {\left\{V^{\left(t\right)}(x)\right\}}_{t=0}^{T_{r}}.$$

Eq. (B2) can be discretized in time using the Euler method as

(B3)
$$\left(V^{\left(t+\Delta t\right)}(x)|\theta \right)=\left(V^{\left(t\right)}(x)|\theta \right)+\Delta t{ℱ}_{nn}\left(t,V^{\left(t\right)}(x)|\theta \right),$$

where the mean and the variance of the Gaussian random variable V will be updated as

(B4a)
$$\left(\mu_{K1},\Sigma_{K1}^{2}\right)=\left(\mu_{{ℱ}_{nn}},\Sigma_{{ℱ}_{nn}}^{2}\right)={ℱ}_{nn}\left(t,\left(\mu_{V^{t}},\Sigma_{V^{t}}^{2}\right)|\theta \right),$$

(B4b)
$$\left(\mu_{V^{t+\Delta t}}|\mu_{V^{t}},\theta \right)=\mu_{V^{t}}+\Delta t\mu_{K1},$$

(B4c)
$$\;\;\left(\Sigma_{V^{t+\Delta t}}^{2}|\Sigma_{V^{t}}^{2},\theta \right)=\Sigma_{V^{t}}^{2}+\Delta t^{2}\Sigma_{K1}^{2},$$

Similarly, Eq. (B2) can be discretized in time using the Runge-Kutta 4th-order method as

(B5a)
$$\left(\mu_{K1},\Sigma_{K1}^{2}\right)={ℱ}_{nn}\left(t,\left(\mu_{V^{t}},\Sigma_{V^{t}}^{2}\right)|\theta \right),$$

(B5b)
$$\left(\mu_{K2},\Sigma_{K2}^{2}\right)={ℱ}_{nn}\left(t+0.5\Delta t,\left(\mu_{V^{t}}+0.5\Delta t\mu_{K1},\Sigma_{V^{t}}^{2}+0.25\Delta t^{2}\Sigma_{K1}^{2}\right)|\theta \right),$$

(B5c)
$$\;\left(\mu_{K3},\Sigma_{K3}^{2}\right)={ℱ}_{nn}\left(t+0.5\Delta t,\left(\mu_{V^{t}}+0.5\Delta t\mu_{K2},\Sigma_{V^{t}}^{2}+0.25\Delta t^{2}\Sigma_{K2}^{2}\right)|\theta \right),$$

(B5d)
$$\left(\mu_{K4},\Sigma_{K4}^{2}\right)={ℱ}_{nn}\left(t+\Delta t,\left(\mu_{V^{t}}+\Delta t\mu_{K3},\Sigma_{V^{t}}^{2}+\Delta t^{2}\Sigma_{K3}^{2}\right)|\theta \right),$$

(B5e)
$$\left(\mu_{V^{t+\Delta t}}|\mu_{V^{t}},\theta \right)=\mu_{V^{t}}+\frac{\Delta t}{6}\left(\mu_{K1}+2\mu_{K2}+2\mu_{K3}+\mu_{K4}\right),$$

(B5f)
$$\left(\Sigma_{V^{t+\Delta t}}^{2}|\Sigma_{V^{t}}^{2},\theta \right)=\Sigma_{V^{t}}^{2}+\frac{\Delta t^{2}}{36}\left(\Sigma_{K1}^{2}+4\Sigma_{K2}^{2}+4\Sigma_{K3}^{2}+\Sigma_{K4}^{2}\right),$$

where $K_{i}$ are Gaussian random variables $K_{i}(x|\theta )∼𝒩(\mu_{Ki}(x,t|\theta ),\mathrm{diag}(\Sigma_{Ki}^{2}(x,t|\theta )))$.

### B2. Discretization in space

Now to discretize the right-hand side of Eq. (B2) in space, we approximate the spatial random state variable $V^{\left(t\right)}(x)$ as a random state variable at a grid point x,

$$V^{\left(t\right)}(x)\approx {\left\{V_{\left(x\right)}^{\left(t\right)}\right\}}_{x=0}^{n_{grid\;}}.$$

Any method can be used to discretize the above equation in space. Here in this study, FDM is used. The discretization results in Gaussian random vector $V(x,t)\approx {\left\{V^{\left(t\right)}(x)\right\}}_{t=0}^{T_{r}}\approx {\left\{{\left\{V_{\left(x\right)}^{\left(t\right)}\right\}}_{x_{0}}^{n_{\mathrm{grid}}}\right\}}_{t=0}^{T_{r}}$. For $2D,V∼𝒩(V|\mu_{V},\mathrm{diag}(\Sigma_{V}^{2}))$, where $\mu_{V}\in R^{n_{t}\times n_{v}\times (n_{x}\times n_{y})}$ and $\mathrm{diag}(\Sigma_{V}^{2})\in R^{n_{t}\times n_{v}\times (n_{x}\times n_{y})}$ are the mean and co-variance vector. Here $n_{t}$, $n_{v}$, $n_{x}$, and $n_{y}$ are the number of time steps, the number of variables, and the number of grid points in the x and y directions, respectively.

For instance, for given $V^{\left(t\right)}∼𝒩(V^{\left(t\right)}|\mu^{\left(t\right)},\mathrm{diag}(\Sigma^{2^{\left(t\right)}}))$, where $\mu^{\left(t\right)}\in R^{n_{v}\times (n_{x}\times n_{y})}$ and $\mathrm{diag}(\Sigma^{2^{\left(t\right)}})\in R^{n_{v}\times (n_{x}\times n_{y})}$ are the mean and co-variance vector, respectively. The discretization of the Laplace term results in

$$\nabla^{2}V_{\left(i,j\right)}^{\left(t\right)}=\frac{V_{\left(i+1,j\right)}^{\left(t\right)}+V_{\left(i-1,j\right)}^{\left(t\right)}-2V_{\left(i,j\right)}^{\left(t\right)}}{\Delta x^{2}}+\frac{V_{\left(i,j+1\right)}^{\left(t\right)}+V_{\left(i,j-1\right)}^{\left(t\right)}-2V_{\left(i,j\right)}^{\left(t\right)}}{\Delta y^{2}}.$$

As the Laplace is a linear operator, we can write

(B6)
$$\nabla^{2}\mu_{\left(i,j\right)}^{\left(t\right)}=\frac{\mu_{\left(i+1,j\right)}^{\left(t\right)}+\mu_{\left(i-1,j\right)}^{\left(t\right)}-2\mu_{\left(i,j\right)}^{\left(t\right)}}{\Delta x^{2}}+\frac{\mu_{\left(i,j+1\right)}^{\left(t\right)}+\mu_{\left(i,j-1\right)}^{\left(t\right)}-2\mu_{\left(i,j\right)}^{\left(t\right)}}{\Delta y^{2}},$$

(B7)
$$\nabla^{2}\text{diag}({\Sigma^{2}}_{\left(i,j\right)}^{\left(t\right)})=\frac{\text{diag}({\Sigma^{2}}_{\left(i+1,j\right)}^{\left(t\right)})+\text{diag}({\Sigma^{2}}_{\left(i-1,j\right)}^{\left(t\right)})+4\text{diag}({\Sigma^{2}}_{\left(i,j\right)}^{\left(t\right)})}{\Delta x^{4}}\;\;+\frac{\text{diag}({\Sigma^{2}}_{\left(i,j+1\right)}^{\left(t\right)})+\text{diag}({\Sigma^{2}}_{\left(i,j-1\right)}^{\left(t\right)})+4\text{diag}({\Sigma^{2}}_{\left(i,j\right)}^{\left(t\right)})}{\Delta y^{4}},$$

where i, j are x, y indices.

## Appendix C. Uncertainty propagation through functions

### C1. Uncertainty propagation through known functions using unscented-transform method

As discussed in the methodology section, the known non-linear operators 𝒦 have been designed to handle probability distribution with the help of the UT method. This is achieved by wrapping the deterministic operator ${𝒦}_{d}$ with the UT function. Detailed procedure is given below.

The UT function takes the mean and variance of the input dynamic variable V and calculates sigma points 𝒳 using the following formulation [57].

$$\begin{array}{ll} & {𝒳}_{0}=\mu_{V},\\ & {𝒳}_{i}=\mu_{V}+{\left(\sqrt{(L+\lambda )\Sigma_{V}^{2}}\right)}_{i},\;i=1,2,\ldots ,L\\ & {𝒳}_{i}=\mu_{V}-{\left(\sqrt{(L+\lambda )\Sigma_{V}^{2}}\right)}_{i-L},\;i=L+1,L+2,\ldots ,2L\\ & W_{0}^{\left(m\right)}=\lambda /(L+\lambda ),\\ & W_{0}^{\left(c\right)}=\lambda /(L+\lambda )+\left(1-\alpha^{2}+\beta \right),\\ & W_{i}^{\left(m\right)}=W_{i}^{\left(c\right)}=1/\{2(L+\lambda )\},\;i=1,2,\ldots ,2L \end{array}$$

**Table:** Algorithm 2. An algorithm for uncertainty propagation through nonlinear function.

Function𝒦(𝒦d,V=(μV,ΣV2),α,β,κ):∣L←dim(μV)λ←α2(L+κ)−LW0(m)←λ∕(L+λ)▷Calculate weightsW0(c)←λ∕(L+λ)+(1−α2+β)𝒳0←μV▷Calculate Sigma pointsfori=1toLdo∣Wi(m)←1∕2(L+λ)▷Calculate weightsWi(c)←1∕2(L+λ)𝒳i←μV+((L+λ)ΣV2)i▷Calculate Sigma points𝒳i+L←μV−((L+λ)ΣV2)i𝒴i←𝒦d(𝒳i)▷Porpagate𝒳through𝒦n𝒴i+L←𝒦d(𝒳i+L)∣endμy←∑i=02LWi(m)𝒴i▷Calculate mean and variable ofo∕pΣy2←∑i=02LWi(c){𝒴i−μy}{𝒴i−μy}Treturnμy,Σy2▷Output random variable∣

where the scaling parameter $\lambda =\alpha^{2}(L+\kappa )-L$ governs the spread of sigma points, with α, κ, and β as tuning hyperparameters. The original UT literature by Wan and van der Merwe [57] suggests using a small positive value for α, and κ=0, β=2 for Gaussian distributions. In our study, we set α=1, κ=0, and β=2, which yield accurate second-moment estimates. To assess the suitability of a given sigma point configuration, one practical strategy is to compare UT-based estimates of mean and covariance against those obtained via sample-based Monte Carlo propagation. This comparison offers a direct empirical mechanism for tuning α, κ, β for improved moment-matching on the function under study. These sigma vectors are thereafter propagated through the nonlinear function ${𝒦}_{d}$,

$${𝒴}_{i}={𝒦}_{d}\left({𝒳}_{i}\right),i=0,1,\ldots ,2L$$

and the mean and covariance for y are approximated using a weighted sample mean and covariance of the posterior sigma points,

$$\begin{array}{ll} & \mu_{y}\approx \sum_{i=0}^{2L}\;W_{i}^{\left(m\right)}{𝒴}_{i},\\ & \Sigma_{y}^{2}\approx \sum_{i=0}^{2L}\;{\;W}_{i}^{\left(c\right)}\left\{{𝒴}_{i}-\mu_{y}\right\}{\left\{{𝒴}_{i}-\mu_{y}\right\}}^{T}. \end{array}$$

Detailed algorithm for operator 𝒦 is given in Algorithm 2.

### C2. Uncertainty propagation through neural-network

Fig. C1 illustrates the Neural Network architectures utilized for learning the unknown functions in both ODE and PDE systems. For ODE-NN, each hidden layer has 8 neurons, while in the case of PDE-NN, 32 neurons were employed. The PDE-NN network follows a point-to-point approach, taking independent distribution statistics (first- and second-order moments) inputs at every point of the grid and generating the corresponding output distribution statistics at that specific grid point.

## Appendix D. Algorithms

The detailed algorithms used to construct a HybridNDiff-UQ solver for Hamiltonian ODEs and Reaction-Diffusin PDEs case are shown in Algorithms 3 and 4, respectively.

**Table:** Algorithm 3. Algorithm for HybridNDiff-UQ solver for Hamiltonian ODEs system.

FunctionHybridNDiff−UQ(xinit=(μx0,Σx0),θ=(θλU,θ𝒩𝒩,θΣx0)):∣t←whilet≤Trdo∣(μs1,Σs12)←𝒩𝒩𝒰r(((μx1,Σx12)t,θ)▷Compute source with UT(μs2,Σs22)←𝒦nr(((μx2,Σx2)t,θ)▷Compute source with NN▷Euler time stepping,(can use RK4)((μxi,Σxi2)t+Δt←((μxi,Σxi2)t+(Δtμxi,Δt2Σsi2)∣endreturn({μxi}t=0Tr,{Σxi2}t=0Tr)▷Predicted time series∣

**Table:** Algorithm 4. An algorithm for HybridNDiff-UQ solver for reaction-diffusion PDEs system.

FunctionHybridNDiff−UQ(Vinit=(μV0,ΣV0),θ=(θλU,θ𝒩𝒩,θΣx0)):∣t←whilet≤Trdo∣(μ∇2Vi,Σ∇2Vi2)←Laplace(μVit,ΣVit),θ)▷Construct Laplace(μs1,Σs12)←𝒦nr((μV1,ΣV1)t,θ)▷Compute source with UT(μs2,Σs22)←𝒩𝒩𝒰r((μV2,ΣV2)t,θ)▷Compute source with NN▷Euler time stepping,(can use RK4)(μVi,ΣVi2)t+Δt←(Δt(θDiμ∇2Vi+μsi),Δt2(θDi2Σ∇2Vi2+ΣVi2))(μVi,ΣVi2)t+Δt←Boundary_Condition((μVi,ΣVi2)t+Δt)∣endreturn({μVi}t=0Tr,{ΣVi2}t=0Tr)▷Predicted time series∣

## Appendix E. Additional computational results

### E1. Hamiltonian

The comparison of HybridNDiff-UQ model prediction’s distribution against HMC distribution for case 1, discussed in Section 3.1.1, is shown in Fig. E1. The results show that the prediction’s distributions are not restricted to Gaussian as the Deep Ensemble strategy has been utilised. Furthermore, the t-SNE projection of the samples of trainable parameters’ posterior distribution captured by the HybridNDiff-UQ is shown in Fig. E2. The plot shows a clear distinction of 10 clusters, which implies the 10 distinct local distributions were captured by the HybridNDiff-UQ method.

In addition to the test cases shown in the Result section, two more cases were conducted on the Hamiltonian ODEs system. Fig. E3 shows the results for the first case where the $f_{1}$ is considered known and $f_{2}$ unknown, therefore NN is tasked to surrogate $f_{2}$. Data for both variables were provided in for 20 s, specifically $𝒟=(x_{1},x_{2}\in {\left(R\right)}^{1}\times [0:0.1:20\;s])$. The mean prediction and the confidence interval are reasonability accurate. In other case, the HybridNDiff-UQ model is trained on the data for the $x_{2}$ variables in 5 to 20 s, specifically $𝒟=(x_{2}\in {\left(R\right)}^{1}\times [5:0.1:20\;s])$. The training process involved 10,000 epochs, and the results obtained are presented in Fig. E4. Due to the limited amount of provided data, the predicted uncertainty is significantly large, except in the region where the data is available. Despite these challenging conditions, the predicted model uncertainty exhibits reasonable behavior. For instance, in the 0 to 5 s region, the model uncertainty for $x_{2}$ remains high, while for $x_{1}$, it increases rapidly.

### E2. Reaction-diffusion

Furthermore, in addition to the test cases discussed in the Results section, we present two additional test cases involving the Reaction-Diffusion PDEs system. Fig. E5 displays the results obtained after training the model for 300 epochs on a 20 × 20 grid with a time-step of 0.01 s. In this case, data for $v_{1}$ and $v_{2}$ is provided up to 3.5 s, represented as $𝒟=(v_{1},v_{2}\in {\left(R\right)}^{20\times 20}\times [0:0.01:3.5\;s])={\left(R\right)}^{20\times 20}\times (3.5:0.01:5\;s])$. Comparing this case to a scenario where only data for $v_{2}$ is provided (Figs. 11 and 12), we observe a reduction in error, improved accuracy in inferring the diffusion coefficients, and accordingly reduction in confidence interval.

Similarly to the test case described in the Corrector Network section, we present the results in Fig. E6, where the training data is provided solely for $v_{2}$. The model is trained for 500 epochs. In the training region, which extends up to 3.5 s, the results exhibit reasonable accuracy. However, as we move into the extrapolation region for $v_{1}$, the predictions deviate from the ground truth. Notably, the predicted uncertainty in the extrapolation region is also high, indicating the lack of reliability in the Hybrid model’s prediction.

## Nomenclature

v: Dynamic state variable
$v_{\theta }$: Dynamic state variable approximated by the deterministic HybridNDiff model for a given θ
$V_{\theta }$: Dynamic state variable distribution with mean $v_{\theta }$, and covariance $\mathrm{diag}(\Sigma_{V,\theta }^{2})$ approximated by the probabilistic HybridNDiff-UQ^,^ model for a given θ
$\mathrm{diag}(\Sigma_{V,\theta }^{2})$: Diagonal covariance matrix of state v approximated by the probabilistic HybridNDiff-UQ model for a given θ
$\mu_{V}$: Mean of state v approximated by the probabilistic HybridNDiff-UQ model considering all θ
$\mathrm{diag}(\Sigma_{V}^{2})$: Diagonal covariance matrix of state v approximated by the probabilistic HybridNDiff-UQ modell considering all θ
